# Unraveling the synergy of radiotherapy and immune checkpoint inhibitors in NSCLC: emerging clinical evidence and novel therapeutic strategies

**DOI:** 10.3389/fonc.2025.1659304

**Published:** 2025-12-03

**Authors:** Khalil-elmehdi Ismaili, Frédérique Fenneteau, Jérémy Bruneau, Miriam Schirru, Hamza Charef, Didier Zugaj, Pierre-Olivier Tremblay, Fahima Nekka

**Affiliations:** 1 Laboratoire de Recherche en Pharmacométrie, Faculté de Pharmacie, Université de Montréal, Montréal, QC, Canada; 2 Clinical Pharmacology, Syneos Health, Québec, QC, Canada; 3 Centre de Recherches Mathématiques, Montréal, QC, Canada

**Keywords:** non-small cell lung cancer, immune checkpoint inhibitors, radiotherapy, immuno-oncology, immunotherapy, clinical trials

## Abstract

Non-small cell lung cancer (NSCLC) remains the leading cause of cancer-related mortality worldwide. While immune checkpoint inhibitors (ICIs) continue to redefine the therapeutic paradigm, their efficacy is limited to a specific proportion of patients. Radiotherapy (RT) is proposed as a strategy to enhance their efficacy, yet its clinical impact remains unclear, hindered by its double-edged sword effect on the immune system across variable settings. This review explores the landscape of RT-ICI combinations in NSCLC, analyzing available evidence in the light of current treatment guidelines. The presented data provide a foundation to validate computational models to predict clinical outcomes and inform tumor-immune dynamics. ClinicalTrials.gov was queried for trials involving both modalities, excluding studies incorporating other therapies except chemotherapy and surgery, other cancer types, or brain metastases. Of the 309 trials identified, 23 met the inclusion criteria, encompassing resectable (n=3), early-stage (n=3), locally advanced (n=10), and advanced NSCLC (n=7). In the neoadjuvant setting, the combination achieves a remarkable pathological response without significantly affecting surgical outcomes. Long-term survival benefit remains elusive. In early-stage unresectable tumors, ICIs are poised to replace chemotherapy as the preferred peri-radiation systemic treatment to prevent recurrences. Current data on locally advanced NSCLC confirm the feasibility of early ICI introduction, chemotherapy-free regimens, and individualized RT approaches. A definitive risk-benefit balance has yet to be established. In advanced stages, while the abscopal effect is well documented, statistical significance remains a concern, necessitating adequately designed studies powered to identify subpopulations most likely to benefit from the combination. Innovative, feasible approaches include RT and dual ICI, re-irradiation beyond progression, multisite micro-radiation, or partial irradiation of large tumors to activate a “hot” tumor microenvironment. In conclusion, while the combination of RT and ICI holds promise, significant challenges remain. A deeper understanding of immune dynamics is crucial. Additionally, the complexity of trial design, coupled with a lack of statistical significance in most available data, underscores the need for more phase 3 trials, the development of powerful biomarkers, and complementary approaches, such as virtual clinical trials, to accelerate progress and refine treatment strategies.

## Introduction

### Epidemiology of NSCLC

Non-small cell lung cancer (NSCLC) is the second most prevalent cancer in the world, with over 2.2 million new cases and 1.8 million deaths in 2020 (1). The modest prognosis, reflected by a 5-year relative survival rate of 28% (2) in the United States and 22% in Canada (3), warranted a surge in clinical trials testing different combinations to improve the efficacy of available treatments.

Though the past century has witnessed remarkable progress in cancer treatment, surgery, chemotherapy (CT), and radiotherapy (RT) continue to represent indispensable pillars of solid tumor management. An expanding repertoire of treatments, including molecularly targeted agents, anti-angiogenic therapies, and antibody–drug conjugates, have proven highly effective in specific contexts, while numerous novel modalities are currently under active investigation (4). The emergence of immunotherapy has driven a new paradigm, shifting from targeting the tumor to empowering patients’ immune systems to counter the tumor avoidance of immune destruction, a mechanism that has been established as a hallmark of cancer since 2011 (5).

### Brief history and mechanisms of action

#### Immune checkpoint inhibitors

The discovery of immune checkpoint inhibitors dates to the 1980s. The interaction between T cell receptors (TCR) and major histocompatibility complex (MHC)-associated peptides on antigen-presenting cells was first identified as the key mechanism of the adaptive immune system. The concept of co-stimulation was introduced with the discovery of CD-28, an immunoglobulin on the surface of T-cells responsible for the amplification of the TCR-MHC activation signal. Conversely, coreceptors that generate negative signaling to dampen effective immune cells are responsible for tumor immune evasion. Molecules that bind either to the cancer cell or the immune cell to prevent this downregulation are referred to as immune checkpoint inhibitors (ICI).

##### CTLA-4

Cytotoxic T lymphocyte antigen 4 (CTLA­4) was the first cloned ICI in 1987. Its primary use as a drug, abatacept, was in 1992, to treat auto-immune rheumatoid arthritis. Its inhibitor, Ipilimumab, was developed by James P. Allison in 1994, with the hope of inventing a universal cancer treatment. Indeed, this idea paved the way to a new era of immuno-oncology, granting him the 2018 Nobel Prize in Physiology or Medicine (6).

CTLA-4 inhibitors have been demonstrated to have different possible sites and mechanisms of action (7, 8). In secondary lymphoid organs around the tumor site, antigen presenting cells (APCs) bind to naïve T cells through the interaction between MHC-bound antigens and T cell receptors (TCRs). The priming of T cells is regulated by the interaction between co-stimulatory (ex. CD28) and inhibitory checkpoints (ex. CTLA-4) and their ligands on the APCs. Therefore, CTLA-4 prevents uncontrolled expansion of activated T cells, favoring the expansion of regulatory T cells (Tregs) over helper T cells, thus generating a tumor immunosuppressive effect (9).

Ipilimumab is considered a turning point in cancer treatment owing to its unprecedent long term impact on survival in advanced melanoma since its first approval in 2011. Combined with nivolumab, its use extended to different indications like renal cell and hepatocellular carcinoma (9, 10).

**Figure 1:**
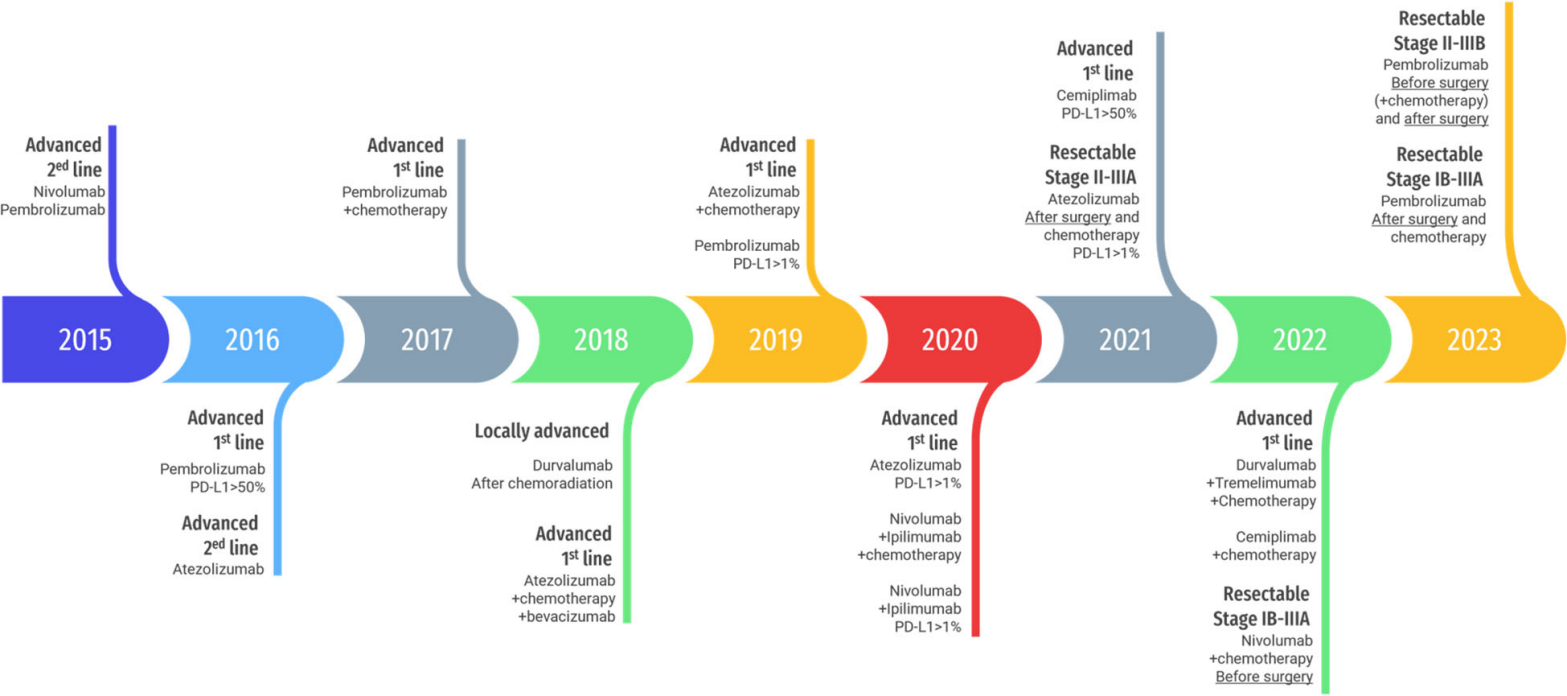
Timeline of FDA approval of immune checkpoint inhibitors for NSCLC. Template by Slidesgo (www.slidesgo.com) and Freepik (*www.freepik.com*).

Regarding NSCLC, anti-CTLA4 monotherapies are shadowed by the higher efficacy and tolerability of programmed death 1 (PD1) and programmed death ligand 1 (PD-L1) inhibitors. However, the combination of the two ICI is approved, with or without chemotherapy, depending on patient’s PD-L1 status. **Figure 1** displays the progression of FDA approvals of ICI for NSCLC.

##### PD-1/PD-L1

When a T cell receptor (TCR) recognizes an antigen bound to the MHC, the co-receptor PD-1 on the surface of the immune cell, notably on cytotoxic T cells (CTL), binds to its ligand PD-L1 on different cells of the tumor microenvironment (TME), mainly on cancer cells’ surface, to trigger an inhibitory signaling to diminish T cells cytotoxic activity. Thus, their blockade by anti-PD1/L1 monoclonal antibodies can counter one of the major tumor immune evasion mechanisms (7).

Pembrolizumab and nivolumab were the first approved PD-1 treatments for second line advanced melanoma in 2014. The repertoire of anti-PD1 (cemiplimab, toripalimab, tislelizumab, dostarlimab) and anti PD-L1 (atezolizumab, durvalumab, avelumab) has then expanded to cover a broader spectrum of indications.

The introduction of ICI doubled the median survival of patients with advanced NSCLC. 5-years survival rate is estimated to be 2-4% without treatment, 15% with classical chemotherapy and radiotherapy, and 20-30% since the introduction of ICI (11, 12). These outstanding results put ICI on the pedestal of treatment guidelines. However, primary and secondary resistance remain a strenuous issue, forcing the scientific community to explore different strategies combining multiple therapies.

##### Adverse events

As immune checkpoints are involved in various immunoregulatory functions such as T-cell priming and peripheral tolerance to self-antigens (13), their inhibition creates an imbalance between cytotoxic and regulatory immune activity, leading to immune-related adverse events (irAEs). Preclinical experiences showed that CTLA-4 −/− mice die prematurely due to supranormal uncontrolled T cell activity (14). irAEs are most frequently dermatological, gastrointestinal, endocrine and hepatic (15). In NSCLC, pneumonitis is the primary concern. These side effects can be mild, reversable through treatment withhold and/or systemic steroid and immunomodulatory therapy, as they can be fatal, requiring intravenous immunoglobulin or plasmapheresis (16). Cases of newly developed type I diabetes have also been reported (17). More than 60% of patients develop irAEs with ipilimumab (15). Grade 3–4 side effects were present in 22% with nivolumab, 28% with ipilimumab and 52% with the combination of both in the Checkmate-067 trial (18).

### Radiotherapy

Radiotherapy is an effective local treatment that offers an alternative to surgery. Since the first use of X-ray in cancer in 1896, radiotherapy underwent accelerated series of improvements featuring administration technologies, imaging technologies and biological understanding (19). These discoveries enabled the delivery of high-dose irradiation specifically to malignant tissues, minimizing damage to surrounding organs through various approaches such as Intensity Modulated Radiation Therapy (IMRT), Image Guided RT (IGRT), 3D Conformal RT (3D-CRT), Stereotactic Body RT (SBRT), proton beam radiotherapy, as well as internal radiotherapy (e.g., brachytherapy).

#### Mechanism of action

Ionizing radiation predominantly targets the DNA, causing single-strand or double-strand breaks, the latter being more difficult to repair and more liable to error-prone repair. This damage can also be indirect through the generation of reactive oxygen and nitrogen radicals. Cell death occurs either during mitosis or secondary to programmed cell death triggered by extensive DNA damage, metabolic alterations, and extracellular signals (20). Tumor cells are more susceptible to mitotic death or senescence due to their rapid division (7).

#### Immunological effect of RT

Besides its effect on tumor cells, radiotherapy has an impact on the tumor vasculature and other components of the tumor microenvironment, preeminently affecting immune cells, and triggering a complex immune response. This response can manifest as both immunosuppressive and immunostimulatory, modulated by the RT protocol of administration (dose, scheduling, radiation type and technology) and the specific clinical setting. Different research teams have reported seemingly contradictory effects. The overall immunological impact of RT can only be assessed under specific conditions, warranting biological investigations in conjunction with clinical trials (21).

A concise overview of the documented mechanisms driving these effects is presented herein.

##### Immunosuppressive effects of RT

Beyond direct effect on tumor cells, radiotherapy-induced DNA damage also impacts lymphocytes. Radiotherapy-induced lymphopenia (RIL) is a critical concern, associated with poorer prognosis (22), and has been reported with an incidence as high as 89% (23). Local lung radiation has the potential to reach and damage radiosensitive bone marrow stem cells (24, 25). Furthermore, other secondary lymphoid organs, such as the spleen and the thymus, are indirectly susceptible to collateral damage through circulating irradiated cells, leading to a reduction of hematopoietic stem cells (26, 27). RT targeting positive lymph nodes can also impair their immune function. Circulating mature lymphocytes in the tumor microenvironment, although accounting for a small portion of total body lymphocytes, are considered the primary cause of lymphopenia and should be treated as an integral organ at risk during RT (28, 29). Indeed, lymphocytes are highly radiosensitive, with DNA damage observable at radiation doses as low as 0.5 Gy. For instance, it is estimated that 99% of circulating blood receives at least 0.5 Gy with conventionally fractionated RT to the glioma. Given the high vascular perfusion in the thoracic region, this poses a significant concern in the context of NSCLC (30). Therefore, precise low-fractionated radiotherapy can be less lethal to total body lymphocytes.

Additional mechanisms contribute to the immunosuppressive effects of RT. It is well established that RT upregulates the expression of immune checkpoints on tumor cells surfaces. Moreover, RT releases immunosuppressive mediators, including adenosine, vascular endothelial growth factor A, transforming growth factor-β (TGF-β) and hypoxia-inducible factor 1-α (HIF-1α). These mediators lead to the polarization of M2 tumor-associated macrophages (TAMs), the recruitment of regulatory T (Treg) cells and myeloid derived suppressor cells (MDSCs), and the inhibition of dendritic cell (DC) maturation (7, 31).

##### Immunostimulatory effects of RT

It is hypothesized that the immunostimulatory properties of radiotherapy may offset its immunosuppressive effects, resulting in a net positive impact on the immune response and supporting a potential synergy with immunotherapies. Radiation-induced cell death releases damage-associated molecular patterns (DAMPs) such as adenosine triphosphate (ATP), heat shock proteins (e.g., HSP70), calreticulin, and high mobility group protein 1 (HMGB1). These DAMPs, presented on the cell surface, act as “eat me” signals, directly activating cytolytic natural killers (NK) cells, inducing the expression of pro-inflammatory cytokines like type 1 interferons (IFNs), interleukin-6 (IL-6) and tumor necrosis factor-α (TNF-α), and promoting the chemotaxis and maturation of APCs, ultimately leading to T cells activation (7, 31). Newly released tumor antigens are presented in the draining lymph nodes, promoting the proliferation of tumor-specific T cells. These effector T cells are subsequently released into the circulation and can target both micro- and macroscopic, irradiated and non-irradiated tumor lesions (32). These responses constitute a fundamental component of the immunogenic cell death elicited by radiotherapy.

This *in situ* cancer vaccine effect is known as the abscopal effect (latin etymology: “ab” away from, “scopus” target) (33). Mole et al. observed the regression of tumors outside the irradiated field in 1953 (34). Until 2014, approximately one case report per year documented this effect (35). Since the approval of ICI, this prevalence increased significantly (36, 37).

RT can also contribute to tumor vascular normalization, mitigating aggressive immunosuppressive tumor phenotype under hypoxia conditions and enhancing the recruitment of tumor-infiltrating lymphocytes (TILs) (38, 39).

These concepts offer a promising avenue in cancer treatment. Radiotherapy can generate an immune response that is amplified by ICIs, potentially achieving significantly improved outcomes. Moreover, a substantial proportion of patients exhibit immunologically ‘cold’ tumors that are unresponsive to ICI. The increased expression of PD-L1 following RT can potentially overcome treatment resistance, converting non-responsive patients (primary resistance) into potential candidates. Treatment beyond progression under ICI is gaining increasing momentum. Theoretically, the addition of RT in this context could prolong response duration and potentially overcome secondary resistance.

## Methodology

**Figure 2:**
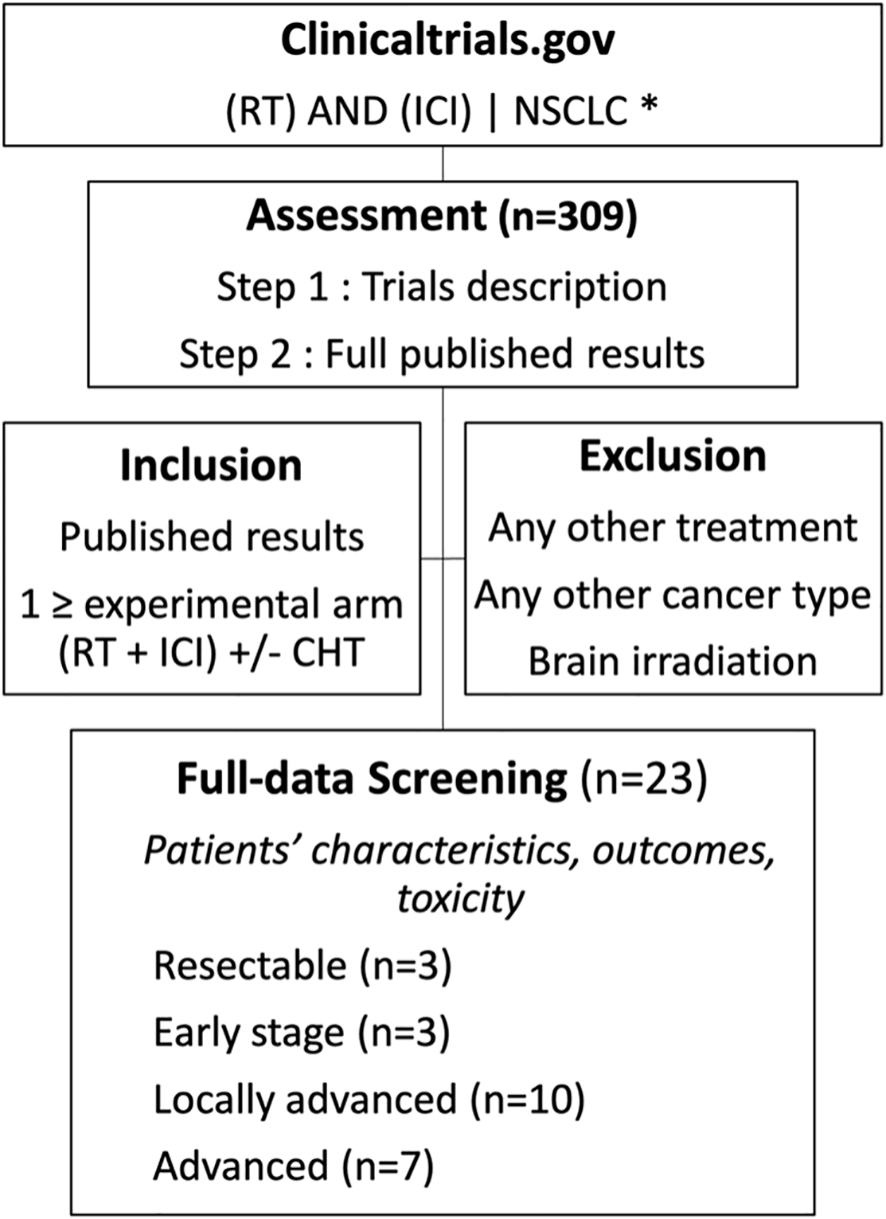
Selection of articles included in the review. * The exact keywords used are:NSCLC(“radiation” or “radiotherapy”) and (PD-L1 OR PD1 OR Checkpoint OR nivolumab OR pembrolizumab OR Atezolizumab OR Durvalumab OR Avelumab OR Cemiplimab OR CTLA 4 OR CTLA OR Ipilimumab OR tremelimumab OR LAG-3 OR Relatlimab).

To assess the feasibility and impact of the RT-ICI combination in NSCLC, we systematically reviewed clinical trials registered in the United States National Library of Medicine (NLM) online database, “clinicaltrials.gov”. Our search criteria included terms referring to ICI and RT with a focus on NSCLC (**Figure 2**). We included trials evaluating the combination of RT and at least one ICI in at least one study arm. Trials involving any additional therapy, except chemotherapy, were excluded. Similarly, trials recruiting patients with indications other than NSCLC were excluded. The initial database extraction was performed in July 2023 and updated in February 2024. Trials results were extracted from the clinicaltrials.gov, as well as from published papers and abstracts, mainly through the collection of oncology clinical trial database “clin.larvol.com”. Available information regarding the studied populations and outcomes is analyzed in this article across 4 clinical categories: resectable tumors, early-stage unresectable tumors, locally advanced unresectable tumors, and advanced disease.

From the 322 trials retrieved from the search on the NLM database search, 24 met our inclusion criteria and had published results. In the following section, we discuss these findings in the context of the National Comprehensive Cancer Network (NCCN) Guidelines to provide a clinical understanding and application of combined RT and ICI across the four clinical scenarios.

## Results

### Resectable tumors

#### Current practice

**Figure 3:**
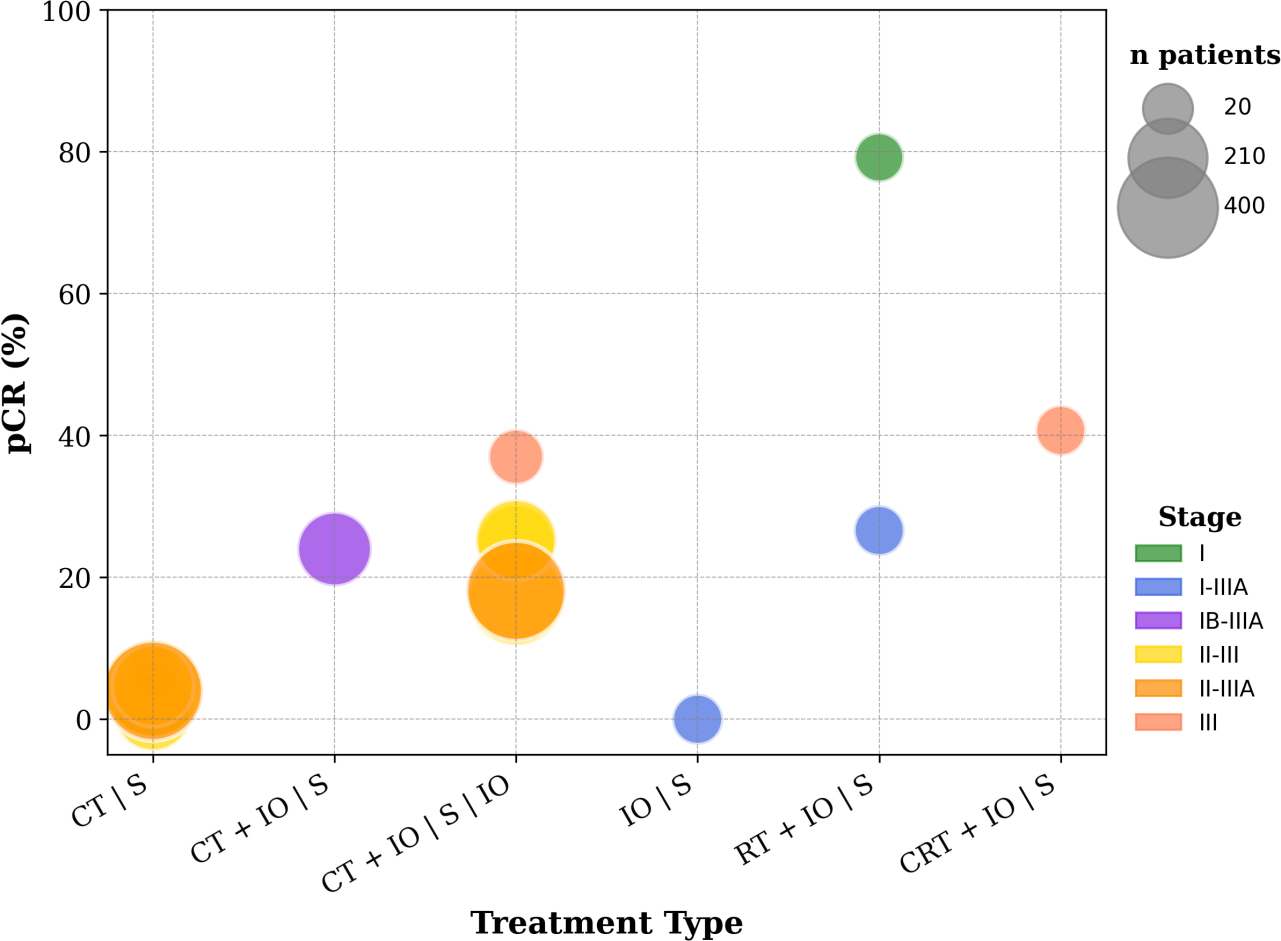
pCR rates from various trials evaluating ICI with or without CT, RT or CRT, in resectable NSCLC. The clinical trials presented in this figure include both those identified through the methodology of this review and the key trials that established the neoadjuvant use of ICI. Included trials: NCT04271384, NCT02904954, NCT03694236, CheckMate 816 (110), NADIM II (111), AEGEAN (112), NEOTORCH (112), CheckMate 77T (113), Impower010 (114), KEYNOTE-671 (43).

When feasible, surgery remains the most effective curative radical local treatment for resectable NSCLC. However, 5-year mortality post-surgery can reach up to 39% (40). Adjuvant therapy is offered to patients with high recurrence risk to prevent regional or distant recurrence and address possible occult micro-metastases. Neoadjuvant treatment aims to facilitate resection, minimize residual tumor burden, and generate systemic anti-tumor immunity by initiating immune priming (*in-situ* vaccine effect). The success of neoadjuvant treatment is often measured by pathological response, i.e., the presence of cancer cells in the resected tissue, which is an informative surrogate biomarker for survival (41). To achieve significant rates of pathological complete response (pCR), ICI need to be combined with CT or RT, as portrayed by **Figure 3**. Theoretically, administering ICI after complete tumor resection provides limited antigen exposure and, consequently, insufficient T-cell activation. By contrast, the addition of radiotherapy to neoadjuvant ICI facilitates early mobilization of the immune system. RT is arguably the treatment most capable of enhancing pathological response; however, the predictive validity of this endpoint remains contested, particularly in light of phenomena such as pseudoprogression. The MISSILE (42) trial illustrated these limitations, showing that RT alone failed to achieve the anticipated pCR rates (60% vs. historic 90%) and did not translate into improved long-term survival. In contrast, in the KEYNOTE-671 (43) and CHECKMATE-77T (44) trials, neoadjuvant ICI-CT has yielded meaningful clinical benefit despite modest pCR rates (18% and 25.3%, respectively), allowing the approval of neoadjuvant pembrolizumab or nivolumab with chemotherapy. These considerations create an opportunity for ICI–RT combinations to demonstrate superiority over ICI–CT. The 3 trials included in this section are reported in **Tables 1**, **2**.

**Table 1:** Description of clinical trials evaluating immunotherapy-radiotherapy combinations in resectable NSCLC.

NCT	Research hypothesis	Objectives	Phase	Design	no. of patients	Stage	Sequence	ICI dosing	RT dosing	Start date	Status	Ref
NCT04271384	Combining preoperative stereotactic ablative radiotherapy (SBRT) with anti-PD-1 immunotherapy will significantly increase the pathological complete response rate in patients with stage 1 NSCLC.	To determine the pathological complete response of the combination of SBRT plus nivolumab as neoadjuvant treatment in early-stage NSCLC.	2	Single arm open label trial of neoadjuvant concomitant SBRT and nivolumab (iSBRT) followed by surgery 12 weeks from day 1 of treatment for patients with NSCLC up to 4 cm.	25	I	RT + IO | S	Nivolumab: 360 mg every 21 days x 3 cycles	SBRT (3 x 18 Gy or 5 x 10 Gy or 8 x 7.5 Gy)	February 12, 2020	Completed	(45, 46)
NCT02904954	Previous phase 2 trials of neoadjuvant anti-PD-1 or anti-PD-L1 monotherapy in patients with early-stage NSCLC have reported major pathological response rates in the range of 15-45%. Evidence suggests that stereotactic body radiotherapy might be a potent immunomodulator in advanced non-small-cell lung cancer (NSCLC).	To find out the effectiveness of the durvalumab with or without SBRT as treatment for stage I, II, and IIIA NSCLC prior to surgery and one year following surgery.	2	Single-center, open-label, **randomized**, controlled, trial of neoadjuvant durvalumab with and without concurrent SBRT.	60	I-IIIA	IO | S RT + IO | S	Durvalumab: 1120 mg every 3 weeks x 2 cycles	SBRT (3 x 8 Gy)	December 2, 2016	Completed	(47–49)
NCT03694236	Adding Durvalumab to neoadjuvant chemoradiotherapy in stage II/III resectable NSCLC could increase the complete pathologic response rate, disease-free survival, and overall survival.	To identify the change of immune signature in tumor microenvironment of NSCLC patients after Durvalumab to identigy a potential biomarker.	1b	Prospective, single center, single-arm, open-label, trial of neoadjuvant concurrent chemoradiation plus durvalumab followed by surgery and adjuvant durvalumab	30	III	CRT + IO | S | IO	Durvalumab: 1500mg every 4 weeks x 2 cycles then for 1 year after surgery	45 Gy in 25 fx	February 12, 2019	Recruiting	(51)

The bold text in the tables indicates subtitles and highlights randomized trials.

**Table 2:** Patients and outcomes description of clinical trials evaluating immunotherapy-radiotherapy combinations in resectable NSCLC.

NCT	Stage (%)	Treatment	Arm	N	Median age(years)	Male (%)	Smokers (%)	Squam. histol.(%)	Median tumor size (mm)	PD–L1 <1% (%)	ECOG1* (%)	OS (%)	PFS or DFS (%) [95% CI]	Response rate (%) [95% CI]	MPR(%) [95% CI]	pCR (%) [95% CI]	Downstage (%) [95% CI]	Grade 3+ AE (%)	Ref
NCT04271384	I	RT + IO | S	EXP	25	68	-	88	–	24.5	–	–	1-year 84%	**EFS** 1-year 84	–	83.3 [61.8-94]	79.2 [57—92]	–	–	(45, 46)
NCT02904954	IA 10% IB 27% IIA 3% IIB 13% IIIA 47%	IO | S	CTRL	30	70.5 (mean)	53.3	80	37	35 (30.5–53.8)	50	30	–	**PFS** 1-year 96 2-years 69 3-years 69 **DFS** 1-year 92 2-years 67 [50–84%] 3-years 63 [46–80.4%]	CR 0 * PR 3.3 SD 80 PD 10 Pseudo-PD 6.7 ORR 3.3 DCR 83.3	6.7 [0.8–22.1]	0	19.2	G3–4 17% G5 3%	(47–49)
NCT02904954	IA 3% IB 23% IIA 20% IIB 13% IIIA 40%	RT + IO – S	EXP	30	69.6 (mean)	50	87	40	45 (31.0–59.8)	20	23	–	**PFS** 1-year 100 2-years 92 3-years 83 **DFS** 1-year 86 2-years 80 [66-94] 3-years 67 [49.6–83.4]	CR 0 PR 46.7 SD 50 PD 3.3 Pseudo-PD 0 ORR 46.7 DCR 96.7	53.3 [34.3–71.7]	26.6	65.4	G3–4 20% G5 3%	(47–49)
NCT03694236	III	CRT + IO | S | IO	EXP	30	65.5	67.0	–	56.7	–	–	–	1-year 85.4%	**DFS** 1-year 79	**ORR** 50 [31.3–68.7%]	74.1 [53.7–88.9]	40.7 [22.4 – 61.2%]	70.4 [49.8–86.2%]	10	(51)

The bold text in the tables indicates subtitles and highlights randomized trials.

#### Reviewed trials

Resectable tumors provide a valuable opportunity to investigate the local effects of radiotherapy and ICI. G. Schvartsman et al. (NCT04271384, phase I, n=25) (45, 46) explored the combination of nivolumab at 360 mg every 3 weeks for 3 cycles, with SBRT at 50–60 Gy in 3–5 fractions over 2 to 3 weeks, followed by surgery 12 weeks from day 1 of treatment, for patients with tumors up to 4 cm without nodal involvement. They reported a notable pCR of 79.2% and a major pathological response (MPR) of 83.3%. Further biological analysis is warranted particularly for the 4 patients who did not achieve MPR, to elucidate the mechanisms of resistance.

The addition of radiotherapy in the peri-operative setting carries inherent safety risks, potentially complicating surgical procedures and affecting postoperative survival outcomes. Benjamin Lee et al. (NCT02904954, phase 2, n=30/30) (47–49) addressed these concerns by combining low-dose focal SBRT (24 Gy in 3 fractions) with 2 cycles of neoadjuvant durvalumab. Their results showed a favorable time to surgery of 2.1 weeks, no added complexity to subsequent resections, no rise in postoperative pneumonitis or pneumonia and no increase in morbidity or mortality, even with 35% of patients undergoing bilobectomy or pneumonectomy. The combination achieved the primary endpoint of a superior MPR, compared to ICI monotherapy (53.3% vs 6.7%, odds ratio 16, p<0.0001). The relatively modest MPR observed in this study may be explained by the moderate RT dose and the high proportion of patients with stage II and III disease. Nonetheless, the 3-year PFS outcomes were encouraging. Although no statistically significant difference was demonstrated at 3 years, the divergence between the two groups becomes apparent beyond 12 months and warrants further investigation.

Tumor downstaging prior to surgery is a clear indicator of neoadjuvant treatment efficacy. For some patients with invasive T3-T4 and N2-N3 disease, concurrent chemoradiotherapy (cCRT) is necessary to reach this goal. However, the role of surgery in this context remains uncertain, as evidenced by the randomized Intergroup-0139 trial (50), which demonstrated improved local response, but lacked a clear survival benefit, potentially due to post-operative complications. The integration of ICI into this multimodal approach was investigated by Jiyun Lee et al. (NCT03694236, phase 1b, n=30) (51). While the promising pCR/MPR rates (40.7%/74.1%) observed encourage further research, caution is advised despite reports of manageable toxicity and absence of perioperative mortality or morbidity.

### Early-stage unresectable tumors

#### Current practice

Curative radiotherapy, with a preference for SBRT (e.g. 24 Gy in 3 fx, 50 Gy in 4 fx, 60 Gy in 5 fx, over 1 to 2 weeks), is widely adopted as the most effective approach for unresectable stage I tumors, providing 2-year local control rates of 90% to 95% and a 2-year OS rates of 50% to 60% (52). Adjuvant chemotherapy has shown limited efficacy and is generally reserved for high-risk stage II patients. While ICIs are well-studied in advanced and locally advanced tumors, their role in early-stage unresectable tumors is yet to prove a favorable risk-benefit ratio, despite the need to improve survival beyond the current state. Herein, we review trials investigating the potential of ICIs to be safely added to SBRT, aiming to reduce distant recurrences and extend survival. The 3 trials included in this section are reported in **Tables 3**, **4**.

**Table 3:** Description of clinical studies evaluating immunotherapy-radiotherapy combinations in early-stage unresectable NSCLC.

NCT	Research hypothesis	Objectives	Phase	Design	no. of patients	Stage	ECOG	Sequence	ICI dosing	RT dosing	Start date	Status	Ref
NCT03148327	The combination of RT and durvalumab will improve PFS in patients with early-stage NSCLC treated with radiation therapy, who are inoperable or who refuse surgery.	To evaluate the safety, tolerability, and efficacy of combining SBRT with durvalumab in patients with early-stage, medically inoperable NSCLC.	1 - 2	Multicenter, prospective trial designed as a phase 2 study with phase 1 safety lead-in, combining durvalumab and SBRT (prefered) or HFRT	18	I-IIA	0-1	ICI x1 5 +/- 3 days | RT - ICI x4	Durvalumab 1500 mg Q4w	SBRT: 54 Gy in 3 fx SBRT: 50 Gy in 4 fx HFRT: 65 Gy in 10 fx	October 11, 2017	Terminated	(53)
NCT02599454	The interplay between radiation and the immune system to promote tumor cell killing will be safely enhanced by the delivery of modern SBRT in combination with atezolizumab, resulting in better local tumor control, the eradication of systemic micrometastasis and ultimately an increase in the cure rate for patients with inoperable early-stage NSCLC.	To assess the side effects and best dose of atezolizumab that can be given together with SBRT in treating patients with stage I NSCLC that cannot be removed by surgery.	1	Multi-institutional phase I study with an expansion cohort testing the addition of six cycles of atezolizumab to SBRT in high-risk, medically inoperable, early-stage NSCLC	20	I-IIIA	0-2	ICI x 3 24 -48h | RT | ICI x3	Atezolizumab 3 mg/kg or 10 mg/kg or 1200 mg (MTD)	SBRT: 50 Gy in 4–5 fx SBRT: 54 Gy in 3 fx	April 26, 2018	Active, not recruiting	(54)
NCT03110978	Combining SBRT with nivolumab may enhance the antitumor effects of either treatment alone and improve clinical outcomes among patients with stage I, selected stage IIa or isolated lung-parenchymal recurrent NSCLC.	To assess the impact of combining immunotherapy with SBRT on regional and distant metastatic progression, compared to SBRT alone, in patients with stage I NSCLC or isolated lung parenchymal recurrence. To characterize the immune responses induced by I-SBRT in contrast to SBRT alone, and its association with clinical outcomes.	2	Open-label, multicenter, **randomized** trial comparing SBRT to SBRT plus Nivolumab	156	I-IIA	0-2	RT | 0-36h | ICI x4	Nivolumab 480 mg, Q4W, 12 weeks	SBRT: 50 Gy in 4 fx SBRT: 70 Gy in 10 fx	June 26, 2017	Active, not recruiting	(55)

The bold text in the tables indicates subtitles and highlights randomized trials.

**Table 4:** Patients and outcomes description of trials evaluating immunotherapy-radiotherapy combinations in early-stage unresectable NSCLC.

NCT	Stage	Treatment	Arm	no. of patients	Median age (years)	Male ratio (%)	Smokers (%)	Squamous histology (%)	Median Tumor Size (range)	PD–L1<1% (%)	ECOG 2 (%)	Median time to follow up (months)	% OS	% PFS or EFS (range)	Local control (%)	Grade 3+ AE (%)	Ref
NCT03148327	I–IIA T1 72% T2 28%	RT + IO	EXP	18	79 (57 – 96)	61.0	89.0	17.0	GTV: 7.9 mL (0.6–270)	–	0*	36 (8.4 – 56.4)	1-year 94.4% 2-year 88.9% 3-year 76.5%	**PFS** 1-year 94.4% 2-year 83.3% 3-year 71.8%	1-year 100% 2-year 93.8% 3-year 94.4%	33.3	(53)
NCT02599454	I–IIIA T1 60% T2 40%	RT + IO	EXP	20	76 (62.2 – 88.9)	45.0	85.0	35.0	Diam: 2.4 cm (1.1–4.4)	62	15	25.8 (7.6 – 41)	**Median** NR 1-year 94.6% 2-year 78.2% 3-year 59.5%	**PFS** Median 25.9 months (95% CI 16.2, NR) 1-year 94.6% 2-year 59% 3-year 37.9%	–	–	(54)
NCT03110978	I–IIA	RT	CTRL	75	72 (66 – 78)	45.0	91.0	19.0	GTV: 4.2 mL (2.4–9.1) Diam: 1.7 cm (1.3–2.2)	45	9	33 (28.7 – 38.1)	–	**EFS** 1-year 83.6% 2-year 63.2% 3-year 55.6% 4-year 53% (42 – 67)	4-years 86.7%	0	(55)
NCT03110978	I–IIA	RT + IO	EXP	66	72 (66 – 75)	30.0	89.0	17.0	GTV: 6.4 mL (2.5–15.1) Diam: 2 cm (1.4–2.6)	41	6	33 (28.7 – 38.1)	–	**EFS** 1-year 93.2% 2-year 83% 3-year 76.1% 4-year 77% (66 – 91)	4-years 100%	15	(55)

The bold text in the tables indicates subtitles and highlights randomized trials.

#### Reviewed trials

Originally designed as a phase II study with an initial phase I safety lead-in, the clinical trial reported by Trudy C. Wu et al. (NCT03148327, n=18) (53) was prematurely terminated due to insufficient patient accrual. The grade 3 or higher (G3+) pneumonitis rate of 16.7%, while higher than the known rate attributable to ICI (1.8%) and SBRT (<10%) monotherapies, remained within acceptable toxicity thresholds for combined therapies. Notably, this trial demonstrated that 5 cycles of durvalumab and 54 Gy SBRT can achieve 93.8% 2-year local control rate and a remarkable 88.9% OS rate.

In a phase 1 trial (NCT02599454, n=20) (54), with a 3 + 3 dose escalation design, reported by Arta M. Monjazeb et al., atezolizumab was escalated to 6 cycles of the therapeutic dose, with 54 Gy SBRT delivered between the 3^rd^ and 4^th^ cycle. No G3+ pneumonitis cases were reported; only one patient (5%) experienced local progression after a median follow-up of 25.8 months, with a 2-year OS of 78.2%. The low PFS (2-years PFS: 59%) can be attributed to the high rate of PD-L1 negative patients (62%), the relatively high representation of tumors over 3cm (40%), and more importantly, the fragility of the population, as demonstrated by an ECOG 2 rate of 15%, which led to a high incidence of death from intercurrent illness without tumor progression.

The only randomized trial identified in this setting (NCT03110978, phase 2, n=66 + 75) (55) compared SBRT (50 Gy in 4 fx or 70 Gy in 10 fractions) with or without 4 cycles of 480 mg of nivolumab every 4 weeks. The addition of the ICI achieved a 62% reduction in the risk of recurrence, disease progression, or death. The reduction of the distant recurrence rate from 16% to 3% confirms the strategic value of combining local and systemic treatments. Moreover, the synergy between the two treatments can also be reflected by the reduction of the 4-year local progression risk from 13% to 0%. No G3+ pneumonitis occurred during the combination treatment and only 10% patients had immune related adverse events (irAE).

Notably, the PD-L1-negative population was well represented in both trials, comprising 40-45% of participants. The success of the protocol may reflect RT-induced PD-L1 upregulation, enhancing responsiveness to ICI. Although not routinely assessed, post-RT PD-L1 testing could validate this hypothesis and refine patient selection. The absence of nodal involvement, which limits the irradiation-induced immunossupressive disruption of the immune cycle, and the ability to administer higher RT doses to small tumors, are factors favoring the manifestation of the synergy.

This approach seems likely to be incorporated into clinical guidelines in the near future, once its role fully established, following ongoing phase III randomized trials (ex. PACIFIC-4 (56), KEYNOTE-867 (57), SWOG/NRG S1914 (58)).

### Locally advanced unresectable tumors

#### Current practice

A quarter of NSCLC patients present with tumors spreading locally to critical tissues and lymph nodes, which limits the feasibility of surgery (59). The need of combining local and systemic therapy is more pronounced. At this stage, the severity and prognosis of the disease spans a broad spectrum between subcategories, with 5-year OS ranging from 2% to 42% (52). Historically, a substantial body of evidence demonstrated that concurrent platinum-doublet chemotherapy and 60 Gy traditionally fractionated radiotherapy, despite an increased but manageable toxicity risk, remains the most effective approach with 5-year survival rates reaching up to 32% (52).

Researchers are striving to optimize these results. Increasing the dose of radiotherapy to 74 Gy led to a poorer median OS (20.3 vs. 28.7 months) in the RTOG 0617 trial (60). The use of new technologies such as intensity-modulated radiation therapy (IMRT), or proton therapy, for its known Bragg peak effect and rapid dose falloff beyond the tumor, can minimize healthy tissues exposure to RT, and allow the administration of higher ablative doses (61).

While SBRT is the standard approach for stage II patients, its application can pose risks in certain cases of LA-NSCLC. The NRG LU008 trial (62) is exploring the addition of SBRT to the primary tumor prior to conventional chemoradiation for nodal lesions. Similarly, the HyCRT-SBRT trial (63) supports incorporating a 35 Gy SBRT boost following 40 Gy hypo-fractionated CRT (85.7% local control).

**Figure 4:**
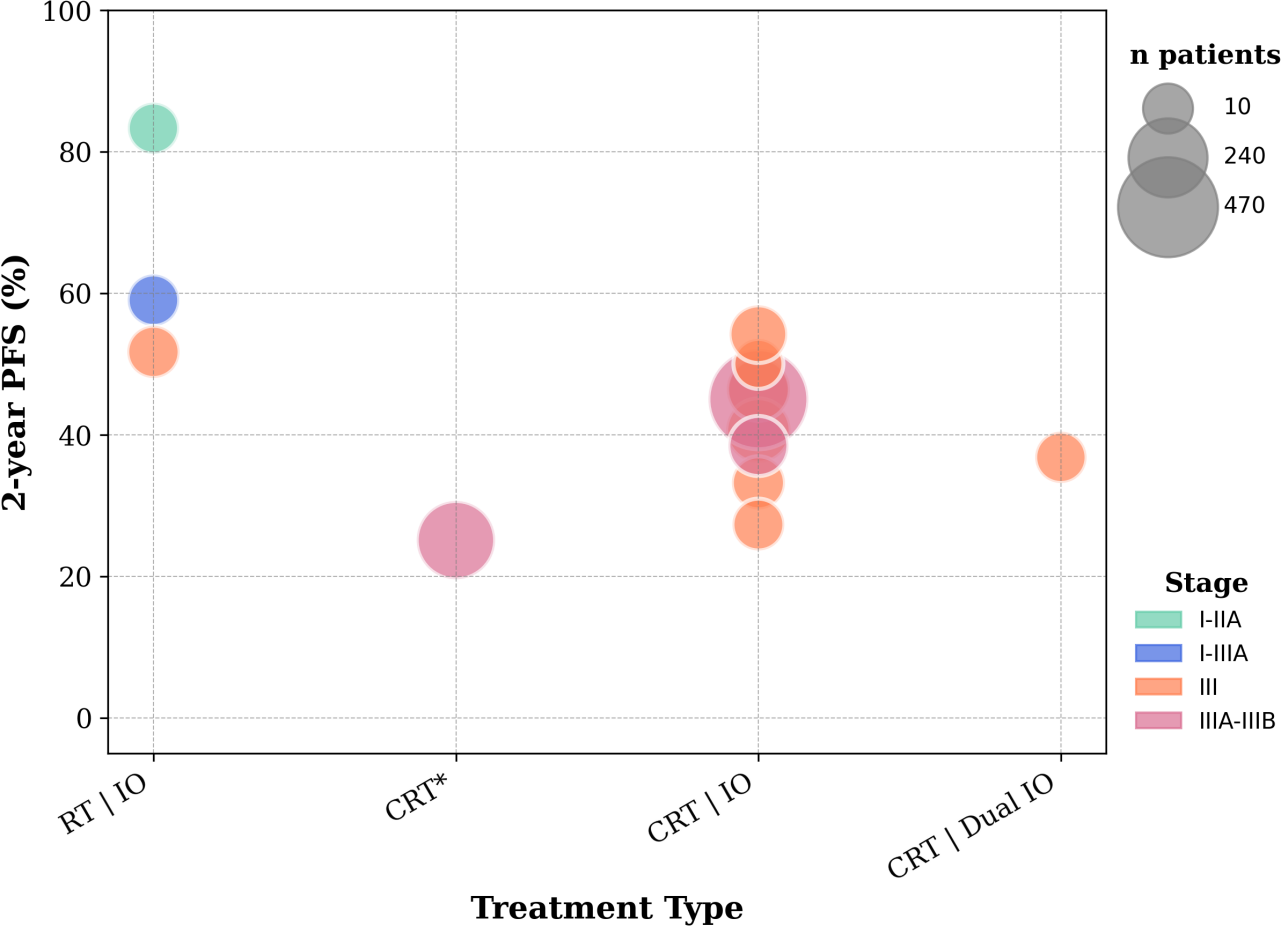
2-year PFS rates from included trials evaluating early-stage and locally advanced unresectable NSCLC. *Patients in the CRT arm received a placebo following previous successful CRT before recruitment.

**Figure 5:**
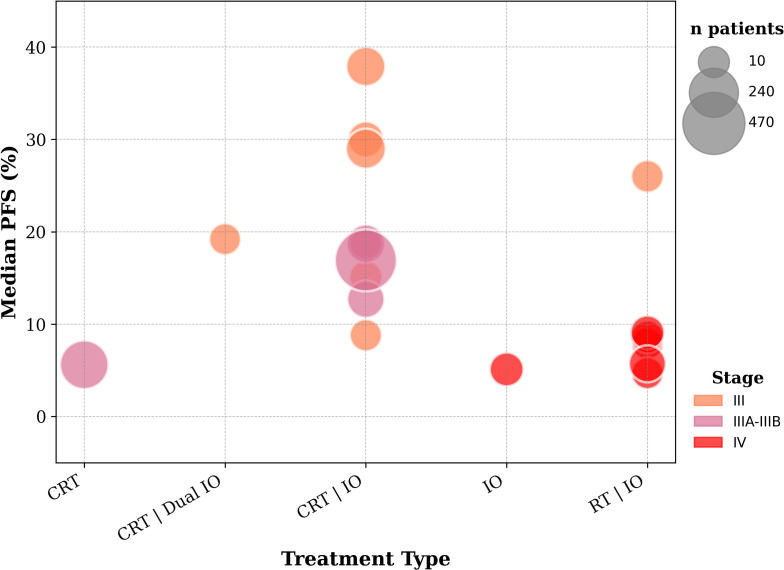
Median PFS from included trials evaluating locally advanced and advanced NSCLC.

No additional benefit was observed from consolidation therapies, until the introduction of durvalumab, after successful c-CRT, through the PACIFIC trial. Two years PFS rates under the ICI-CRT combination range between 20% and 60% in the reviewed trials. These results breathed new life into the field, directing subsequent studies toward innovative approaches to further harness ICI potential in LA-NSCLC. The 10 trials included in this section are reported in **Tables 5**, **6**. The 2-years PFS rates and median PFS are displayed in **Figures 4**, **5**.

**Table 5:** Description of clinical trials evaluating immunotherapy-radiotherapy combinations in LA-NSCLC.

NCT	Research hypothesis	Objectives	Phase	Design	no. of patients	Stage	Sequence	ICI dosing	RT dosing	CT dosing	Start date	Status	Ref
NCT02125461 PACIFIC	Based on preclinical evidence suggesting that chemotherapy and radiotherapy may up-regulate PD-L1 expression in tumor cells, durvalumab may provide clinical benefit after chemoradiotherapy in unresectable stage III NSCLC patients	To assess the effects of durvalumab following concurrent CRT in patients with Stage III unresectable NSCLC	3	**Randomized** placebo-controlled trial with a 2:1 ratio, testing Durvalumab as consolidation therapy 1 to 42 days after successful CRT	713	III	CRT | IO	Durvalumab 10 mg/kg every 2 weeks for up to 12 months	54 to 66 Gy	Two or more cycles of platinum-based chemotherapy (containing etoposide, vinblastine, vinorelbine, a taxane [paclitaxel or docetaxel], or pemetrexed)	May 7, 2014	Completed	(64, 65)
NCT02343952 LUN14–179	Consolidation therapy with pembrolizumab following concurrent chemoradiation may improve overall survival and delay disease progression in patients with unresectable or inoperable stage IIIA or IIIB NSCLC.	To evaluate whether consolidation therapy with pembrolizumab following concurrent chemoradiation prolongs the time to distant metastatic disease in patients with inoperable or unresectable stage III NSCLC.	2	Single-arm, multi-institutional trial testing pembrolizumab as consolidation therapy 28 to 56 days following successful CRT	92	III	CRT | IO	Pembrolizumab 200mg every 3 weeks for up to 12 months	59.4-66.6 Gy	Cisplatin/Etoposide, Cisplatin/Pemetrexed or Carboplatin/Paclitaxel	March 2015	Completed	(66)
NCT02621398	Giving pembrolizumab together with paclitaxel, carboplatin, and radiation therapy may kill more tumor cells in patients with stage II-IIIB NSCLC.	To determine the safety and tolerability of PD-1 inhibition concurrently with definitive chemoradiotherapy for NSCLC.	1	Multicenter trial escalating from sequential to concomitant pembrolizumab and CRT in a 3 plus 3 design	21	III	CRT | IO CRT + IO	Pembrolizumab every 3 weeks Cohort 1: 200 mg, day 56 - 84 Cohort 2: 100 mg day 29 Cohort 3: 200 mg, day 29 Cohort 4: 100 mg, day 1 Cohort 5: 200 mg, day 1	60 Gy in 30 fx	weekly carboplatin (AUC = 2) and paclitaxel (50 mg/m2)	April 11, 2016	Active, not recruiting	(67)
NCT03631784 KEYNOTE–799	Within each platinum doublet chemotherapy cohort, the percentage of participants who develop Grade 3 or higher pneumonitis is ≤10% and objective response rate (ORR) by blinded independent central review (BICR).	To evaluate safety and efficacy of pembrolizumab in combination with cCRT in patients with unresectable Stage III NSCLC.	2	Global, open-label trial testing concomitant pembrolizumab plus cCRT	216	III	CRT + IO | IO	Pembrolizumab 200 mg on day 1 every 3 weeks for 14 weeks	60 Gy in 30 fx	Pembrolizumab 200 mg on day 1 every 3 weeks for 14 weeks	October 19, 2018	Completed	(68, 69)
NCT02525757 DETERRED	Adding immunotherapy concurrently with CRT would increase efficacy without significant additive toxicity in patients with Stage III unresectable NSCLC.	To evaluate safety/toxicity and feasibility of combining atezolizumab with cCRT followed by consolidation full dose carboplatin/paclitaxel with atezolizumab for 2 cycles and then maintenance atezolizumab for 1 year.	2	Two parts trial escalating from sequential to concomitant atezolizumab and CRT, both followed by the ICI.	40	IIb-III	Part 1: CRT | CT + IO | IO Part 2: CRT + IO | CT + IO | IO	Atezolizumab 1200mg every 3 weeks	60–66 Gy in 30–33 fx	CRT: weekly carboplatin (AUC 2.0) and paclitaxel 50 mg/m2. 3 weeks after CRT: carboplatin (AUC 6.0) and paclitaxel 200 mg/m2	January 26, 2016	Active, not recruiting	(71, 72)
NCT0243408 NICOLAS	Concurrent administration of nivolumab with standard chemoradiotherapy may improve PFS and OS in patients with unresectable stage III NSCLC	To investigate the tolerability and the efficacy when nivolumab is added to cCRT to patients with unresectable advanced stage IIIA/​B NSCLC.	2	Single arm trial testing concomitant chemoradiation and nivolumab followed by the ICI	79	IIIA-IIIB	CT | CRT + IO | IO	Nivolumab 360 mg every 3 weeks for 4 cycles followed by 480 mg every 4 weeks for up to 1 year	66 Gy in 33 fx	Cisplatin/Carboplatin: 3 cycles combined with either vinorelbine, etoposide, or pemetrexed (nonsquamous histologic subtype)	November 25, 2015	Completed	(73)
NCT03102242 AFT–16	Integrating atezolizumab as a neoadjuvant (pre-CRT) and adjuvant (post-CRT) therapy may enhance clinical outcomes in patients with unresectable stage III NSCLC	To evaluate the safety and efficacy of administering atezolizumab before and after CRT in patients with unresectable stage III NSCLC		Single arm trial testing 4 cycles of neoadjuvant, and adjuvant atezolizumab with CRT	62	III	IO | CRT | IO	Atezolizumab 1200 mg every 3 weeks for up to 1 year	60 Gy in 30 fx	Carboplatin AUC = 2 + paclitaxel 50 mg/m2 q 7 days x 6 weeks concurrent with radiation Consolidation Carboplatin AUC = 6 + paclitaxel 200 mg/m2 q 21 days x 2 cycles beginning 3–5 weeks after completion of radiation.	November 01, 2017	Completed	(74)
NCT03523702 SPRINT	A personalized and de-intensified chemotherapy-free treatment approach could improve outcomes for selected patients with LA-NSCLC	To explore if, for locally advanced NSCLC patients whose tumors have high levels of PD-L1, a combination of immunotherapy and a personalized 4-week radiotherapy course could be more effective than cCRT	2	Single arm trial testing 3 cycles of pembrolizumab before risk-adapted RT followed by consolidation pembrolizumab	25	II-III	IO | RT | IO	Pembrolizumab 200 mg every 21 days	55–48 Gy in 20 daily fx over 4 weeks	–	August 30, 2018	Completed	(78)
NCT03663166	Addition of concurrent ipilimumab with chemoradiotherapy followed by consolidative nivolumab would be safe and tolerable for patients with unresectable stage III NSCLC	To determine if Stage III NSCLC patients treated with ipilimumab with thoracic radiation therapy followed by nivolumab monotherapy every 4 weeks for up to 12 months show an improved 12-month PFS rate compared with a 12-month historical PFS rate of 49% among patients treated in a similar fashion with concurrent chemoradiotherapy	1 | 2	Multi-institution trial testing concurrent ipilimumab with CRT followed by maintenance nivolumab	19	III	CRT + IPI | NIVO	Ipilimumab 1 mg/kg week 1 and 4 Nivolumab 480 mg every 4 weeks for up 12 cycles starting 1–3 weeks after CRT	60 Gy in 30 fx	Cisplatin and etoposide (Arm A), carboplatin and paclitaxel (Arm B), or cisplatin and pemetrexed (Arm C)	November 20, 2018	Terminated	(79)
NCT03087760	Reirradiation (reRT) with proton beam therapy (PBT) may offer a chance of cure while minimizing toxicity for patients with isolated intrathoracic recurrences of NSCLC. However, distant failure remains common, necessitating strategies to integrate more effective systemic therapy	To assess the safety, tolerability, and anti-tumor activity of pembrolizumab in patients with recurrent NSCLC after previous radiation therapy	2	Single arm trial testing proton beam reRT for locoregional recurrences followed by pembrolizumab for patients without progression	22	III	RT +/- CT | IO	Pembrolizumab 200 mg every 21 days starting on week 4 to 12, for up to 12 months	60 Gy (18 to 70), 2 Gy (1.8 to 4) per dose	No information	January 18, 2017	Completed	(83, 84)

The bold text in the tables indicates subtitles and highlights randomized trials.

**Table 6:** Patients and outcomes description from clinical trials evaluating immunotherapy-radiotherapy combinations in LA-NSCLC.

NCT	Stage (%)	Treatment	Arm	N	Median age (year)	Male (%)	Smokers (%)	Squam. histol. (%)	PD–L1<1% (%)	PD–L1>50% (%)	Median time to follow up (months)	Median OS (months)	OS (%) (Range or 95% CI)	Median PFS (months)	PFS (%) (range or 95% CI)	Response rate timing (months)	ORR and DCR (%)	Response rate (%)	G3+ and SAE (%)	Ref
NCT02125461 PACIFIC	IIIA 52.7 IIIB 45.1	CRT	CTRL	237	64 (23–90)	70.0	91.1	47.1	24.47	–	34.2 (0.7–74.7)	29.1 (95% CI: 22.1 to 35.1)	**1-year:** 74.6% (CI: 68.5 to 79.7) **2-year:** 55.6% (48.9 to 61.8) **3-year:** 43.6% (CI: 37.1 to 49.9) **5-year:** 33.4% (27.3 to 39.6)	5.6 (4.6 to 7.8)	**1-year:** 35.3% (29.0 to 41.7) **2-year:** 25.1% (19.3 to 31.2) **5-year:** 19% (13.6 to 25.2)	Best response after a median 14.5 months (0.2 to 29.9)	**ORR:** 16 (11.3–21.6) **DCR:** 71.9	**CR:** 0.5 **PR:** 15.5 **SD**: 55.9 **PD:** 27.7 **ND:** 0.5	**G3+ TR:** 26.1 **SAE**: 23.08	(64, 65)
NCT02125461 PACIFIC	IIIA 52.9 IIIB 44.5	CRT | IO	EXP	476	64 (31–84)	70.2	91	43	18.9	–	34.3 (0.2–74.7)	47.5 (95% CI: 38.1 to 52.9)	**1-year:** 83.1 (CI: 79.4 to 86.2) **2-year:** 66.3 (61.7 to 70.4) **3-year:** 56.7 (CI: 52.0 to 61.1) **5-year:** 42.9 (38.2 to 47.4)	16.9 (13.0 to 18.1)	**1-year:** 55.9 (51.0 to 60.4) **2-year:** 45.0 (40.1 to 49.8) **5-year:** 33.1 (28.0 to 38.2)	Best response after a median 14.5 months (0.2 to 29.9)	**ORR:** 28.4 (24.3–32.9) **DCR**: 81.1	**CR:** 1.4 **PR:** 27.1 **SD:** 52.6 **PD:** 16.5 **ND:** 2.3	**G3+ TR**: 29.9 **SAE**: 9	(64, 65)
NCT02343952 LUN14–179	IIIA 60 IIIB 40	CRT | IO	EXP	92	64.4 (45–84)	64.1	94.6	44.6	20.8	58.5	32.2 (1.2–46.6)	35.8 (24.2 to NA)	**1-year:** 81.1 **2-year:** 62 **3-year:** 48.5	18.7 (12.4 to 33.8)	**1-year:** 61.2 **2-year:** 46.3 **3-year:** 37.4	–			**G3-4**: 56.5 **SAE**: 27.96	(66)
NCT02621398	IIIA 38 IIIB 62	CRT | IO CRT + IO	EXP	23	69.5 (53–85)	48.0	95.0	–	21	26	16 (95% CI 12.0–22.6)	29.4 (26.9 to NA)	**1-year:** 85.2 (70 to 100) **2-year:** 78.6	18.7 (95% CI 11.8 – 29.4)	**1-year:** 69.7 (CI 49.3 – 90.2) **2-year:** 32.4	Best response	**ORR:** 90 **DCR:** 95	**CR:** 16 **PR:** 74 **SD:** 5		(67)
NCT03631784–A KEYNOTE–799	IIIA 36.6 IIIB 56.3 IIIC 7.1	CRT + IO | IO	EXP	112	66 (46–90)	67.9	94.7	65.2	18.8	–	18.5 (13.6–23.8)	35.6 (26.1–44.2)	**1-year:** 81.3 **2-year:** 40.2 (31.1–49.1)	29.0 (16.6–48.5)	**1-year:** 67.1 **2-year:** 40.6 (28.9–52.0)	Best response	**ORR**: 70.5 (61.2-78.8) **DCR**: 88.4	**CR:** 3.6 **PR:** 67 **SD:** 17.9 **PD:** 0.9 **ND:** 10.7	**G3+ TR**: 65.2 **SAE**: 58.93	(68, 69)
NCT03631784–B KEYNOTE–799	IIIA 38.2 IIIB 41.2 IIIC 20.6	CRT + IO | IO	EXP	102	64 (35–81)	60.8	95.1	0	27.5	–	13.7 (2.9–23.5)	NR (41.1–NR)	**1-year:** 87 **2-year**:54.6 (43.9–64.0)	37.9 (17.9–NR)	**1-year:** 71.6 **2-year:** 46.4 (33.7–58.1)	Best response	**ORR:** 70.6 (60.7-79.2) **DCR:** 93.1	**CR:** 4.9 **PR:** 65.7 **SD:** 22.5 **ND:** 6.9	**G3+ TR**: 51.0 **SAE** 45.1	(68, 69)
NCT02525757 DETERRED	IIb 10 IIIa 20 IIIb 60 IIIc 10	CRT | CT + IO | IO	EXP	10	66 (52–71)	90.0	100	70	33	22	39.2	26.5	**1-year**:79.9 **2-year:** 50 **3-year:** 40.1 **4-year:** 40.1	18.9	**1-year:** 60 **2-year:** 50 **3-year:** 39,9 **4-year:** 39.9	–			**G3+**: 80	(71, 72)
NCT02525757 DETERRED	IIb 16 IIIa 40 IIIb 37 IIIc 7	CRT + IO | CT + IO | IO	EXP	30	68 (50–83)	60.0	70	23	26.6	24	39.2	NR	**1-year:** 79.9 **2-year:** 69.7 **3-year:** 59.2 **4-year:** 52	15.1	**1-year:** 56.7 **2-year:** 33.2 **3-year:** 33.2 **4-year:** 29.6	–			**G3**+: 80	(71, 72)
NCT02434081 NICOLAS	IIIA 35.4 IIIB 63.3	CT | CRT + IO | IO	EXP	79	62 (41–78)	67.1	96.2	35.4	–	–	21 (PFS) (IQR 15.8–25.8) 32.6 (OS)	38.8 (95% CI`: 26.8 – NA)	**1-year:** 75.7 (CI: 64.6–83.7) **2-year:** 63.7 (CI: 51.9–73.4)	12.7 (95% CI 10.1 – 22.8)	**1-year:** 53.7 (CI 42.0 – 64.0) **2-year:** 38.4	Best response	**ORR:** 73.4 (62.3–82.7) **DCR:** 86.1	**CR:** 6.3 **PR:** 67.1 **SD:** 12.7 **PD:** 7.6 **ND:** 6.3	**SAE** 48.1	(73)
NCT03102242 AFT–16	III 100	IO | CRT | IO	EXP	62	63.9 (57.5–71.1)	48.4	88.7	–	73.5	–	31.2 (8–40)	NR	**1-year:** 87 (CI: 79 to 95.8) **2-year:** 73.7 (CI: 63.4 to 85.7)	30 (95% CI, 15.8 to na)	**1-year:** 68.9 (CI 68.1–81.6) **2-year:** 54.2 (CI, 42.7to 68.7)	Best response post induction IO	**ORR:** 66.2 (57.3 to 76.1) **DCR:** 77.5	**CR**: 8.1 **PR**: 58.1 **SD**: 11.3	**G3+**: 48.4 **SAE** 53.13	(74)
NCT03523702 SPRINT	II 4 IIIA 52 IIIB 36 IIIC 8	IO | RT | IO	EXP	25	71 (62–77)	52.0	–	44	0	100	22	NR	**1-year:** 92 **2-year:** 76	26	**1-year:** 76 **2-year:** 51.7	Post induction IO	**ORR:** 48 **DCR:** 92	**CR**: 4 **PR**: 44 **SD**: 44 **PD**: 8	**G3+ TR** £ 28	(78)
NCT03663166	IIIA 21.1 IIIB 63.2 IIIC 15.8	CRT | Dual IO	EXP	19	66 (39–76)	52.6	89.4	31.6	–	–	30.1 (95% CI 15.0–35.0)	NR (95% CI: 6.1–NR)	**1-year:** 63 (CI 38-80)	19.2 (95% CI 6.1 - NA)	**1-year:** 58% (CI 33-76) **2-year:** 36.8	Best response	**ORR:** 66.7 (CI 41 to 87) **DCR:** 100	**CR:** 5.6 **PR:** 61.1 **SD:** 33.3 **PD:** 0	**G3+ TR**: 84 **SAE**: 68.42	(79)
NCT03087760	III	RT +/- CT | IO	EXP	22	68 (54–85)	50.0	–	36.4	40.9	13.6	38.7 (95% CI 25.6–NR)	22.8 (6.9 - NA)	**1-year:** 64 (CI: 40–80) **2-year:** 44.2 **3-year:** 39 (CI: 18–58)	8.8 (4.2 - 23.7)	**1-year:** 45 (CI. 24–64) **2-year:** 27.3 **3-year:** 18 (CI: 6%-36%)	–			**G3+**: 45.4 **SAE** 45.445.4	(83, 84)

The bold text in the tables indicates subtitles and highlights randomized trials.

#### Reviewed trials

While the PACIFIC trial (NCT02125461, phase 3, n=476 + 237) (64, 65) improved historical survival outcomes, it remains exclusive to patients who respond to CRT. In excluding patients with tumor progression or G2+ pneumonitis post cCRT, the design of the study was impeccable to target patients with the most likelihood to respond and tolerate the treatment. The delay of 1 to 42 days between CRT and ICI in this sequential regimen further reduced the risk of toxicity. 5-year survival update reported, at a median follow-up of 34.2 months, a largely favorable durable response, reflected by 28% reduction in the risk of death (median OS 47.5 vs 29.1 months) and a 45% reduction in the risk of disease progression or death (median PFS 16.9 vs 5.6 months). Durvalumab scored 12.4 percentage points in the objective response rate (ORR) over placebo (28.4% vs 16%) with 51.1% responding patients having a durable response beyond 5 years. While any grade irAE (24.2% vs 8.1%) was higher with durvalumab, G3+ toxicities were similar between the two groups, which demonstrates a manageable safety profile. Out of the known (63.2%) PD-L1 profiles, 70.2% were positive. Notably, sampling was done pre-CRT and didn’t consider the potential increase in PD-L1 expression post-CRT. The main issue of the study was the lack of stratification for the PD-L1 status, which left room to doubt whether this treatment is effective in PD-L1-negative patients. While the FDA doesn’t discriminate based on the PD-L1 status, the European Medicines Agency approves the PACIFIC protocol for PD-L1-positive patients only.

In a veteran population of 221 PD-L1-positive patients and 119 negative ones, a similar 2-year OS rate was achieved in both groups (72% and 76%). Median OS wasn’t reached in the first group and was 47 months in the latter, which is similar to the PACIFIC trial results.

Pembrolizumab demonstrated similar 1-year PFS/OS (61.2%/81.1%) and G3–4 pneumonitis (5.4%) in the LUN 14–179 trial (NCT02343952, phase 2, n=92) (66). A larger randomized trial can further confirm those results.

Interestingly, *post-hoc* analysis of the PACIFIC trial, examining the delay between cCRT and durvalumab, suggests that early introduction of durvalumab (1 to 14 days vs 14 to 42 days) is more effective in improving PFS. This observation is consistent with the underlying mechanistic rationale of RT–ICI synergy, as earlier introduction of ICI allows greater exploitation of RT-induced T cell mobilization. Thereafter, the scientific community’s interest shifted toward studying the concomitant regimen, potentially expanding patient reach while optimizing treatment efficacy.

The trials reviewed within this paper show a pattern of slightly increased toxicity and no definitive efficacy benefit. It is important to note that these results cannot be compared to the PACIFIC trial, since the latter evaluated safety and efficacy solely after the completion of c-CRT and had very specific patient selection criteria.

The phase 1 NCT02621398 (n=23) (67) trial established the safety of combining pembrolizumab concurrently with CRT in a 3 plus 3 design. Despite promising safety and efficacy results, administering durvalumab concurrently with cCRT, rather than 2+ weeks later, led to similar G3+ pneumonitis rates (8.3% vs 9%) but higher incidence of G2+ pneumonitis (41.7% vs 18.2%), without achieving a higher PFS (1-year PFS 66.7% vs 77.8%). However, the design of phase 1 escalation trials doesn’t allow comparative conclusions, and further exploration can still be pursued. A similar protocol was studied in the Keynote-799 trial (NCT03631784, phase, A. squamous n=112; B. non-squamous n=104) (68, 69), showing very promising median PFS (29 and 45.3 months in group A and B respectively) and OS (35.6 and 56.7 months in group A and B respectively) with reasonable G3+ pneumonitis rate (7.5%) (70).

The DETERRED trial (NCT02525757, phase 2) (71, 72) escalated from sequential cCRT plus adjuvant chemotherapy and atezolizumab (part 1, n=10) to a concurrent protocol (part 2, n=30) without exceeding the toxicity threshold (G3+ pneumonitis 0% and 3% in part 1 and 2 respectively). Based on the manageable rate of immune-related G3+ AE (30% and 20% in parts 1 and 2, respectively) and a 20% discontinuation rate due to toxicity, the authors concluded that the treatment was safe and feasible. However, a high rate of G3+ AE was observed in part 2 (80%), which questions the relevance of adjuvant chemotherapy.

Overall efficacy outcomes were promising (overall median OS 26.5 months, and not reached in parts 1 and 2). The trial wasn’t powered to evaluate the difference between the two groups, nevertheless, it is noteworthy to mention that the concurrent regimen didn’t show superiority over the sequential one in terms of PFS (15.1 vs 18.9 months), especially in PD-L1 negative patients and those with targetable driver oncogene mutations.

The concurrent regimen was also evaluated with nivolumab in the NICOLAS trial (NCT02434081, phase 2, n=79) (73). The interim safety analysis reached a positive conclusion with no G3+ pneumonitis 6 months post radiation in the first 21 patients. The 11.7% rate of G3+ pneumonitis in the total safety cohort wasn’t alarming. The median PFS was 12.7 months. The 1-year PFS rate of 53.7% narrowly missed the anticipated threshold of 60%. The median OS of 32.6 months was particularly encouraging.

Without any definitive proof of an added value of the concurrent schedule, the mechanistic rationale and the reasonable toxicity profile seen in these trials confirms the opportunity to continue exploring this protocol in the experimental setting.

A neoadjuvant approach could also provide broader access to ICI with potentially less toxicity. Four cycles of atezoluzumab were administered in the AFT-16 trial (NCT03102242, phase 2, n=62) (74), before proceeding, in the absence of progression, to c-CRT (n=44), followed by optional consolidation chemotherapy (n=28) and adjuvant atezolizumab (n=35). Despite 73.5% of available samples being PD-L1 negative, neoadjuvant atezolizumab achieved a notable disease control rate (DCR) of 74.2% after 12 weeks of induction treatment. However, almost 30% of enrolled patients did not move to the c-CRT phase, due mostly to disease progression. Further investigation of this subpopulation can reveal whether the delay before definitive c-CRT is critical. Interestingly, the overall population reached 1- and 2-year OS rates of 87% and 73.7%, and PFS rates of 68.9% and 54.2% respectively. The 48.4% rate of G3+ AE is not unexpected due to the combined treatment modalities, yet only 6.4% patients had G3+ pneumonitis and 19.4% discontinued treatment due to adverse events.

Randomized trials are essential before confirming the superiority of the neoadjuvant or concomitant use of ICI compared to the adjuvant sequence. Such effort is currently conducted in trials like the PACIFIC2 (durvalumab) (75), KEYLYNK-012 (pembrolizumab) (76), and ECOG-ACRIN EA5181 (nivolumab) (77).

Following the success of ICI monotherapy in PD-L1 high expressing patients with advanced disease, a shift from chemotherapy as a necessary systemic therapy for LA-NSCLC is rationally possible. The SPRINT trial (NCT03523702, phase 2, n=25) (78) supports the hypothesis that this approach, along with risk adapted de-intensified RT, offers a promising toxicity profile with only 1 (4%) G3+ pneumonitis and no grade 4 or 5 toxicities. Selected patients had a PD-L1 TPS >= 50% with a median of 75%. Owing to an ORR of 48% with only 2 patients progressing after 3 cycles of pembrolizumab, RT was administered at a lower dose (48 Gy instead of 55 Gy) to more than half patients with smaller tumors. Survival outcomes did not seem to be compromised by this more cautious approach (1-year PFS of 76%, 1 and 2-year OS of 92% and 76%, respectively).

This ambitious trial embraced the principle that radiotherapy should not follow a one-size-fits-all approach, particularly for locally advanced NSCLC, which is characterized by significant intra- and inter-patient heterogeneity. For instance, organs at risk are more likely to be irradiated in LA-NSCLC due to the involvement of the mediastinal region and hilar lymph nodes, which limits the use of high tumoricidal RT doses.

Achieving greater synergy can be explored by integrating additional immunotherapies. Building on the approval of combined CTLA-4 and PD-L1 inhibitors plus chemotherapy in advanced disease, ipilimumab was tested concurrently with cCRT, followed by nivolumab (NCT03663166, phase 1|2, n=19) (79), for patients with large tumor volume (median planning target volume 627.9 cc). However, this combination comes at the cost of increased toxicity; 63% of patients discontinued treatment due to adverse events, including 5 (26.3%) grade 5 AE. CTLA-4 inhibition lowers the threshold for radiation induced toxicity, thereby increasing the susceptibility to adverse pulmonary effects, negatively affecting survival outcomes (1-year PFS 58%, 1 year OS 63%, ORR 66.7%).

Could the outcome be different if a CTLA-4 was integrated in a chemotherapy free, RT de-intensified, or in a sequential sequence? It appears unlikely that such avenues will be explored in the near future, with the presence of alternative opportunities of combination therapies with emerging agents, such as oleclumab or monalizumab in the PACIFIC-9 (80) trial.

Tumor recurrence precipitates a steep decline in prognosis. In the PACIFIC trial, most relapses (80.6%) occur intrathoracically (81). Early detection of loco-regional progression offers a chance to containment through re-irradiation of selected lesions; however, the risk of distant recurrence (34%) is substantial (82). Pembrolizumab, adjuvant to proton pencil beam reRT with or without chemotherapy (NCT03087760, phase 2, n=16), achieved better 3-year PFS (18%) and OS (39%) rates compared to previous reRT results (PFS 12%, OS 14.9%), and did not increase G3+ toxicities (45% vs 42%) (83, 84). However, significant differences in patient populations and trial protocols severely restrict the ability to make definitive conclusions from these comparisons. The trial was terminated early due to the widespread adoption of ICI. Indeed, patients who progress after cCRT may be eligible for advanced stages immunotherapy protocols. This experience displays the intricacies of trial design due to the rapid evolvement of clinical practices, where standard of care can quickly become outdated. ICI rechallenge continues to be an active area of investigation. Another exploratory strategy involves re-irradiation concurrently with continuous ICI beyond progression, building on the rationale that many patients on ICI eventually develop progression. The ‘catch-them-all’ approach acknowledges intratumoral heterogeneity, where some tumor clones remain ICI-sensitive while others escape. Delivering RT in this context may enhance local tumor control, expose neoantigens from resistant clones, enhancing the immunoreactivity and accessibility of the TME, and potentiate renewed immune-mediated tumor elimination.

### Advanced stages

#### Current practice

**Figure 6:**
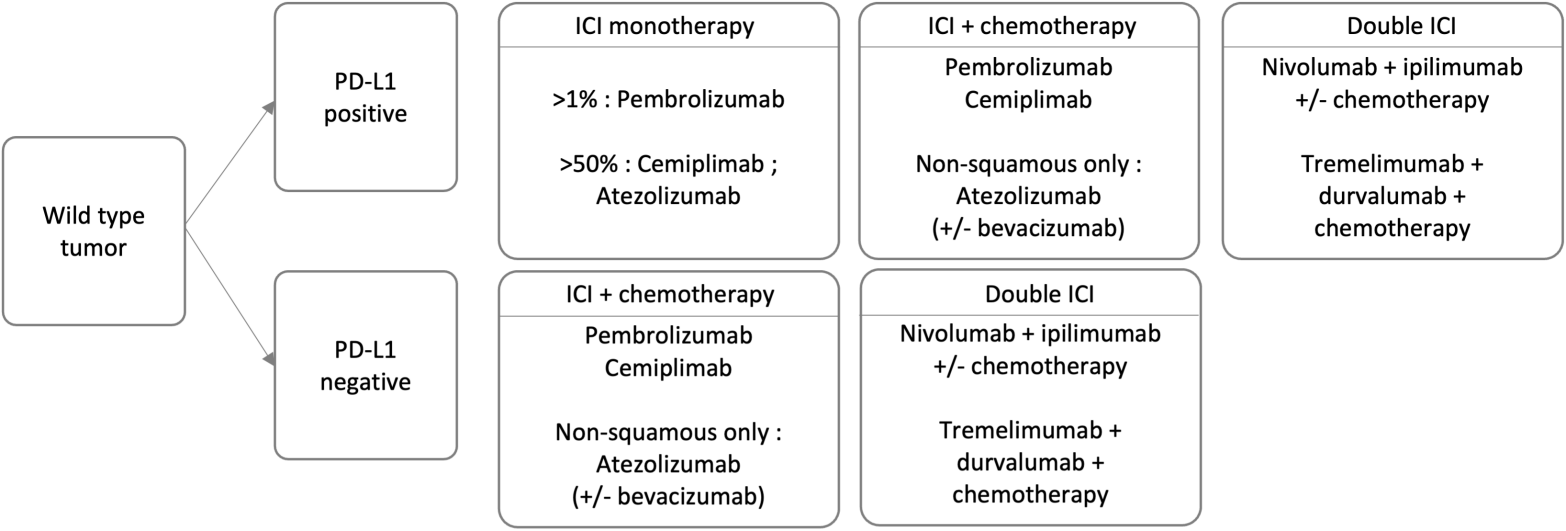
Treatment options for advanced wild type NSCLC.

**Figure 7:**
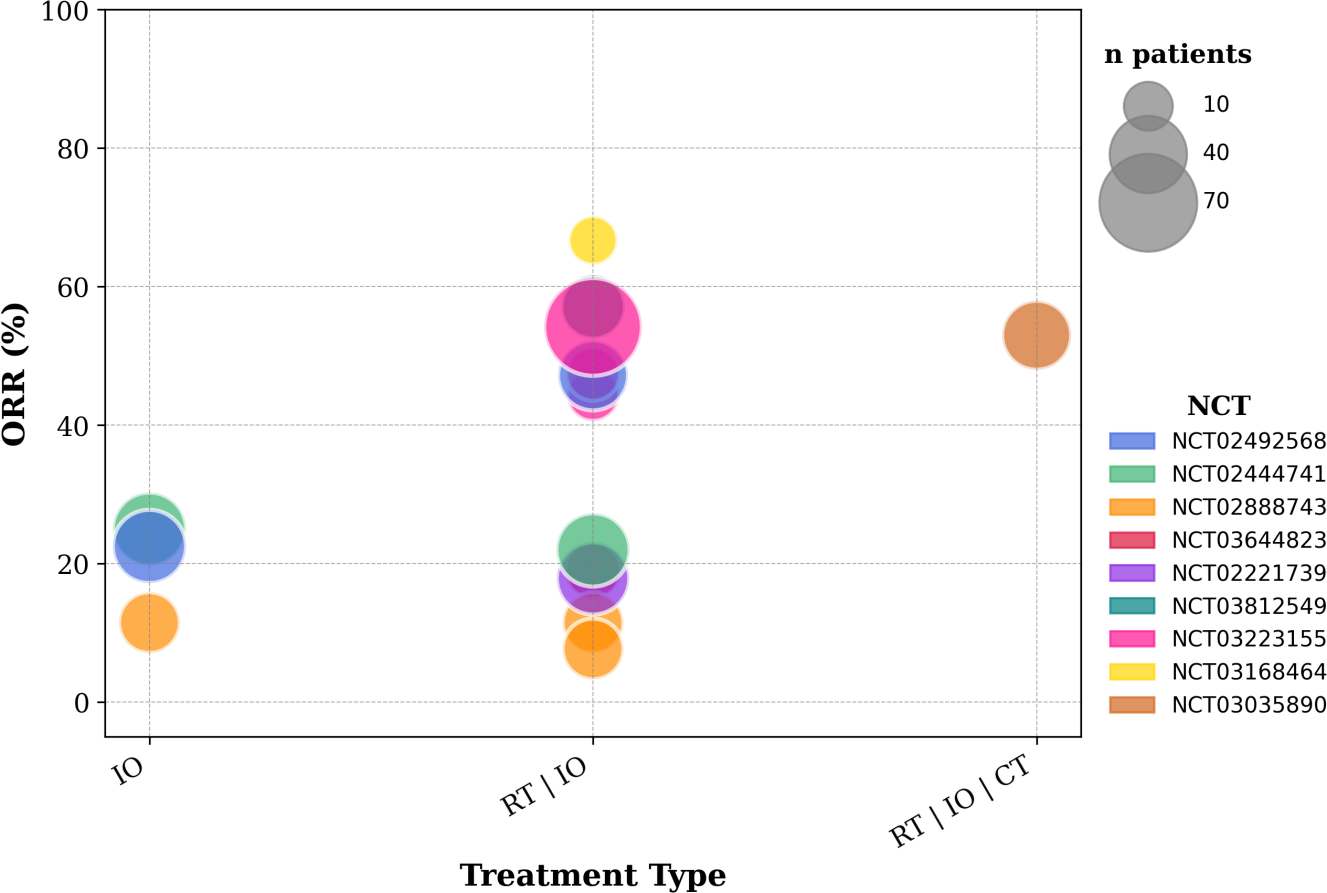
Objective response rates from included trials evaluating advanced NSCLC.

Unfortunately, over 40% of newly diagnosed patients present with advanced-stage disease (85). In selected cases of limited progression, radical surgery or radiotherapy can still be beneficial. Patients with a poor performance status (a key prognostic factor for survival) undergo supportive care or palliative treatment. Extensive research aims to limit progression, prolong survival, and preserve a dignified quality of life. Treatment approaches depend on different factors such as tumor features (e.g. histology, molecular and immune profile) and patients characteristics (e.g. health status, medical history, treatment acceptance). Testing for actionable mutations (e.g. EGFR, ALK, ROS1, BRAF) is an important step to evaluate the use of target agents. Wild-type tumors can benefit from ICI (pembrolizumab, cemiplimab, atezolizumab or the combination of nivolumab and ipilimumab) as first or subsequent line therapy, with the addition of chemotherapy, depending on the PD-L1 status (**Figure 6**). ICIs have proven to be pivotal in improving survival in responding patients. However, almost 80% (86) of patients do not respond to therapy, as depicted by ORRs in **Figure 7**. Attention has shifted to exploring ways to enhance ICI efficacy. Theoretically, the mechanisms of cancer immune evasion, such as the “camouflage” by downregulating the expression of DAMPS and MHC I or the remodeling of the TME (87), can be reversed by RT. Also, the lack of PD-L1 expression, which is the major mechanism of primary resistance to ICI, can be overcome by RT. Overall, besides its palliative use, and its radical use in limited progressions, we review whether the addition of radiotherapy can broaden the range of patients who respond to ICIs and improve their efficacy. The 7 trials included in this section are reported in **Tables 7**, **8**.

**Table 7:** Description of clinical studies evaluating immunotherapy-radiotherapy combinations in advanced NSCLC.

NCT	Research hypothesis	Objectives	Phase	Design	N	Sequence	ICI dosing	RT dosing	Start date	Status	Ref
NCT02492568	In a significant subset of patients with recurrent NSCLC, immunotherapy after SBRT will be superior to treatment with immunotherapy alone and that SBRT, given to a single metastatic site of the tumor, will augment the immune response to the tumor.	To evaluate the increase in ORR in the pembrolizumab alone arm compared to the pembrolizumab after SBRT arm at 12 weeks	1	Multicenter **randomized** trial testing SBRT on a single tumor site preceding pembrolizumab by 0 to 7 days	92	**CTRL**: ICI **EXP**: RT | ICI	Pembrolizumab 200 mg every 3 weeks for up to 24 months	24 Gy in 3 fx on alternate days	July 2015	Completed	(88)
NCT02444741	The combination of pembrolizumab and radiation (either SBRT or CFRT) is tolerable without a high rate of dose limiting toxicity. The combination of pembrolizumab and radiation can lead to tumor regression both within the radiation treatment field and outside the field via the abscopal effect and treating a secondary lesion with low dose radiation improves abscopal response.	To evaluate the safety, tolerability, and efficacy of pembrolizumab with or without radiation therapy in metastatic NSCLC.	1-2	A Phase I 3 + 3 dose-escalation design, followed by a **randomized** Phase II, with pembrolizumab administered alone or concurrently with SBRT or CFRT. Salvage radiation was offered between the second and third cycle of pembrolizumab in case of progression.	72	**CTRL:** ICI | salvage RT **EXP:** ICI + RT | ICI	Pembrolizumab escalated to 200 mg every 3 weeks for up to 32 cycles	50 Gy in 4 fx (SBRT) 45 Gy in 15 fx (CFRT)	September 17, 2015	Active, not recruiting	(89)
NCT03035890	Adding SBRT to ICI in metastatic NSCLC is safe, enhances the systemic anti-tumor immune response, and improves treatment outcomes by modulating the tumor microenvironment.	To determine the safety and efficacy of combined ICI and radiation therapy in ICI-naive metastatic NSCLC patients.	1	Nonrandomized trial combining SBRT with standard-of-care ICI alone (n=19) or with 4 cycles doublet carboplatin/pemetrexed chemotherapy (n=16). Additional SBRT offered in case of progression.	35	ICI +/- CT | RT | ICI +/- CT | ICI	standard-of-care Pembrolizumab, Nivolumab, Atezolizumab	24 to 36 Gy in 3 fx or 30 to 35 Gy in 5 fx between cycles 1 and 2	January 23, 2017	Completed	(90)
NCT03644823	The combination of atezolizumab with SBRT could enhance the systemic anti-tumor immune response and improve outcomes in patients with advanced NSCLC.	To investigate toxicity and efficacy in NSCLC patients (stage III-IV, palliative treated) treated with atezolizumab and radiotherapy.	2	Nonrandomized trial testing concomitant atezolizumab and SBRT in second or later line of non-ICI systemic therapy	21	ICI | RT | ICI	Atezolizumab 1200 mg every 3 weeks for up to 2 years	18 Gy in 3 fx to one or two lesions between the first and second infusion	August 15, 2018	Terminated	(91)
NCT02221739	Local radiotherapy combined with ipilimumab could induce an anti-tumor immune response at the irradiated site, potentially leading to systemic tumor regression through the abscopal effect.	To investigate the efficacy and safety of the combination of radiation therapy and an ipilimumab in the treatment of metastatic NSCLC	2	Nonrandomized trial testing concurrent palliative radiotherapy (IGRT or IMRT) to one metastasis and ipilimumab	39	RT + ICI	Ipilimumab 3mg/kg on day 0, 22, 43, 64.	30 Gy in 5 fx or 28.5 Gy in 3 fx 3	July 1, 2014	Completed	(92)
NCT03812549	Combining low-dose radiotherapy (LDRT) with SBRT and sintilimab will enhance the anti-tumor immune response in patients with stage IV NSCLC expressing PD-L1.	To investigate the safety and tolerability of sintilimab in combination with concurrent SBRT and LDRT in treating patients with stage IV NSCLC.	1	Nonrandomized trial testing SBRT concurrently with LDRT, followed by sintilimab within 7 days after radiation completion, in PD-L1 positive patients. A dose escalation Phase to determine the optimal LDRT dose was followed by a dose expansion Phase.	29	RT | ICI	Sintilimab 200mg every 3 weeks for up to 24 months	30Gy in 3 fx (SBRT) to a small lesion and 4 Gy in 2 fx (LDRT) to a large lesion concurrently	January 18, 2019	Completed	(93)
NCT03223155	SBRT may improve outcomes for stage IV NSCLC patients receiving immunotherapy through both direct cytoreduction and increased immunogenicity.	To evaluate the safety of nivolumab and ipilimumab plus sequential or concurrent multisite SBRT in patients with stage IV NSCLC.	1	**Randomized** trial testing first line SBRT to 1 to 4 isocenters, before (sequential arm) or after (concurrent arm) the first cycle of dual ICI in widely metastatic patients. Expansion non-randomized Phase of the concurrent arm.	75	RT | dual ICI	Nivolumab 3 mg/kg every 2 weeks plus Ipilimumab 1 mg/kg every 6 weeks	30–50 Gy in 3–5 fx (paritial irradiation if size >65 cc)	September 7, 2017	Active, not recruiting	(94)
NCT03168464	Initial local radiotherapy during anti-CTLA-4 blockade with Ipilimumab treatment is safe and increases the ORR to ICI by 20% with the combination of Ipilimumab and Nivolumab. The immune response can be prospectively monitored among the treated patients. Modifications in the stool microbiome of these patients correlate with response to RT-ICI.	To evaluate the safety of combining radiotherapy and ipilimumab as an induction regimen for ipilimumab/nivolumab in NSCLC. To assess its impact on ORR by inducing an *in-situ* vaccine effect, and explore biomarkers of treatment response, including T cell receptor repertoire, serum markers, microbiome changes, PFS, and OS.	1-2	Simon’s two stage optimal design, with >5/15 patients required to respond in stage 1 to proceed to the stage 2, testing concurrent RT plus dual ICI.	15	ICI | RT | dual ICI	**Day 1 +/-24h** Ipilimumab 3 mg/kg **Day 22** Ipilimumab 1 mg/kg every 6 weeks Nivolumab 360 mg every 2–3 weeks	**Day 1:** 30 Gy in 5 fx to one lesion	October 9, 2017	Terminated	(97)
NCT02888743	Immunotherapy with durvalumab and tremelimumab, may induce changes in body’s immune system and may interfere with the ability of tumor cells to grow and spread. Radiation therapy uses high energy x-rays to kill tumor cells and shrink tumors. Giving durvalumab and tremelimumab with radiation therapy may work better in treating patients with colorectal or non-small cell lung cancer.	To investigate the potential benefit of PD-L1 (durvalumab) and CTLA-4 (tremelimumab) inhibition alone or with low-dose radiotherapy or hypofractionated radiotherapy, in patients with metastatic NSCLC who have progressed on previous PD-L1 therapy.	2	An open-label, multicenter, **randomized** trial with a 1:1:1 design, comparing dual ICI with or without LDRT or HFRT.	90	**CTRL:** Dual ICI **EXP:** Dual ICI | RT | ICI	Durvalumab 1500 mg every 4 weeks for up to 13 cycles plus tremelimumab 75 mg every 4 weeks for up to 4 cycles	**LDRT**: 0.5 Gy BID x 2 days x 4 first cycles **HFRT**: 24 Gy in 3 fx every other day during the first cycle only	June 6, 2017	Active, not recruiting	(98)

The bold text in the tables indicates subtitles and highlights randomized trials.

**Table 8:** Patients and outcomes description of clinical studies evaluating immunotherapy-radiotherapy combinations in advanced NSCLC.

NCT	Treatment	Arm	N	Median age (years)	Male (%)	Smokers (%)	Squamous histology (%)	PD-L1<1% OR >50% (%)	ECOG (%)	Median time to follow up (months)	Median OS (months) (range)	Median PFS (%) (months)	PFS (%)	Median DOR (months) (range)	Response rate timing (months)	ORR or DCR (%)	RR (%)	Grade 3+ SAE (%)	Ref
NCT02492568 PEMBRO-RT	IO	CTRL	40	62 (35 – 78)	57.0	>80	10.0	<1%: 66.0 >50%: 13.0	ECOG1: 43 ECOG2: 3	23.6 (0.1 - 34.4)	7.6 (6.0 -13.9)	1.9 (1.7 -6.9)	6 months: 35 12 months: 24 18 months: 20	–	Best systemic response	**ORR:** 22.5 **DCR:**47.5	**CR:** 2.5 **PR:** 20 **SD:** 25 **PD:** 52.5	22	(88)
NCT02492568 PEMBRO-RT	RT + IO	EXP	36	62 (35 – 78)	56.0	>81	14.0	<1%: 50.0 >50%: 28.0	ECOG1: 53 ECOG2: 0	23.6 (0.1 -34.4)	15.9 (7.1 -NR)	6.6 (4.0 -14.6)	6 months: 51 12 months: 34 18 months: 28	–	Best systemic response	**ORR:** 47.2 **DCR:**72.2	**CR:** 8.3 **PR:** 38.9 **SD:** 25 **PD:** 27.7	17	(88)
NCT02444741 MDACC	IO	CTRL	40	65 (52 – 91)	62.5	77.5	27.5	<1%: 25.0 >50%: 22.5	ECOG1: - ECOG2: 0	20.4	–	5.1 (3.4 -12.7)	6-months: 48.5 12-months: 35.9 18-months: 30.6	–	Best systemic response	**ORR:** 25 **DCR:**63.9	**CR:** 0 **PR:** 25 **SD:** 38.9 **PD:** 41.7	25	(89)
NCT02444741 MDACC	RT + IO	EXP	40	65 (52 – 85)	65.0	75	15.0	<1%: 22.5 >50%: 10.0	ECOG1: - ECOG2: 0	20.4	–	9.1 (3.6 -18.4)	6-months: 66.5 12 months: 37.6 18-months: 32.7	–	Best systemic response	**ORR:** 22 **DCR:**63.92	**CR:** 0 **PR:** 22.2 **SD:** 41.7 **PD:** 36.1	30	(89)
NCT03035890	RT + IO +/- CT	EXP	35	66 (58.5 - 70.5)	49.0	91	14.0	<1%: 11.0 >50%: 57.0	ECOG1: 60 ECOG2: 14	14.0 (IQR 5.4 – 23.5)	15.0 (11.0 -40.0)	6.9 (4.3 -26.0)	–	–	Best systemic iRECIST response	**ORR:** 52.9 **DCR:**73.5	**CR:** 5.9 **PR:** 47 **SD:** 20.6 **PD:** 26.5	**SAE** 71.43	(90)
NCT03644823 ComIT-1	RT + IO	EXP	21	61.7	61.9	90.4	4.8	<1%: 71.4 >50%: 4.8	ECOG1: 61.9 ECOG2: 4.8	26.5 (17.6 -35.5)	NR	4.3 (2.2 -8.7)	–	17.8	Best systemic response	**ORR:** 19 **DCR:**57.1	**CR:** 0 **PR:** 19 **SD:** 38.1 **PD:** 23.8 **ND:** 19	14.29	(91)
NCT02221739	RT + IO	EXP	39	68 (48 – 97)	41.0	-	7.7	<1%: >50%:	ECOG1: 82 ECOG2: 17.9	43 (38–47)	7.4 (4.4 -12.6)	3.81 (3.06 -5.49)	6-months: 22.8 12-months: 9.7	–	Systemic response at 2.9 months	**ORR:** 17.9 **DCR:**30.8	**CR:** 5.1 **PR:** 12.8 **SD:**12.8 **PD:** 69	48.72 **SAE:**	(92)
NCT03812549 IHC	RT + IO	EXP	29	-	89.7	-	-	<1%: 0.0 >50%: 34.4	ECOG1: - ECOG2: -	15.5 (1.2 -32.5)	NR	8.6 (5.7 -11.5)	–	13.6 (7.9 -19.3)	–	**ORR:** 57.1 (95CI 37.2-75.5) **DCR:**78.6 (95CI 59.1-91.7)	–	20.7 TR	(93)
NCT03223155 COSINR Concurrent	RT + IO	EXP	18	63.2 (45 – 78)	50.0	77.8	11.1	<1%: 33.3 >50%: 27.8	ECOG1: 38.9 ECOG2: 0	17 (2.2–31.0)	NR	7.9 (3.3-17.8)	6-months: 55 12-months: 40.6 18-months: 24.3	–	Best response	**ORR:** 44.4 **DCR:**72.2	**CR:** 5.6 **PR:** 38.9 **SD:** 27.8 **PD:** 27.9	72.2	(94)
NCT03223155 COSINR Sequential	RT | IO	EXP	19	59.0 (36 – 76)	63.2	89.5	10.5	<1%: 63.2 >50%: 21.0	ECOG1: 52.6 ECOG2: 0	17 (2.2–31.0)	NR	4.7 (3.4–9.4)	6-months: 42 12-months: 25.2 18-months: 12.7	–	Best response	**ORR:** 47.4 **DCR:**52.6	**CR:** 5.3 **PR:** 42.1 **SD:** 5.3 **PD:** 47.4	73.7	(94)
NCT03223155 COSINR (expansion)	RT + IO	EXP	75	65	55.0	-	17.0	<1%: 46.7 >50%: 18.7	ECOG1: ECOG2: 0	–	34 (17 –42)	5.7 (4.4 - 11)	1-year: 35 2-year: 17.7	–	Best Response	**ORR:** 54.2 **DCR:**66.7	**CR:** 5.6 **PR:** 48.6 **SD:** 12.5 **PD:** 33.3	5.3 TR	(94)
NCT03168464 (phase 1)	RT + IO	EXP	9		-	-	-	<1%: >50%: 11.11	ECOG1: - ECOG2: 0	–	–	–	–	–	2.3	**ORR:** 22.22 **DCR:**55.55	**CR:** 11.11 **PR:** 11.11 **SD:** 33.33 **PD:** 22.22 ND 22.22	0 TR	(97)
NCT02888743	IO	CTRL	26	65 (60 – 70)	65.0	-	12.0	<1%: 15.0 >50%: 12.0	ECOG1: 81 ECOG2: 0	12.4 (IQR 7.8 - 15.1)	NR (90CI 4.9 - NR)	3.3 (1.8 - 5.5)	–	NR (10.3 - NR)	Best systemic response	**ORR:** 11.5 (90CI 1.2 - 21.8) **DCR:**30.8 (CI90 15.9 -45.7)	**CR:** 0 **PR:** 12 **SD:** 42 **PD:** 38 ND 8	4 **SAE** TR	(98)
NCT02888743	HFRT + IO	EXP	26	65 (57 – 72)	58.0	-	19.0	<1%: 15.0 >50%: 12.0	ECOG1: 73 ECOG2: 4	12.4 (IQR 7.8 - 15.1)	9.7 (90CI 5.1 - NR)	4.0 (90CI 2.1 - 7.0)	–	NR (2.5 - NR)	Best systemic response	**ORR:** 11.5 (CI90 1.2 - 21.8) **DCR:**34.6 (CI 90 19.3 -50.0)	**CR:** 0 **PR:** 12 **SD:** 38 **PD:** 38 ND 12	15 SAE TR	(98)
NCT02888743	LDRT + IO	EXP	26	65 (60 – 73)	69.0	-	4.0	<1%: 19.0 >50%: 8.0	ECOG1: 65 ECOG2: 0	12.4 (IQR 7.8 - 15.1)	9.1 (90CI 93.8 - 23.9)	4.6 (90CI 2.1 - 7.2)	–	4.9 (4.3 - 5.5)	Best systemic response	**ORR:** 7.7 (CI 90 0 - 16.3) **DCR:**23.1 (CI 90 9·5 - 36·7)	**CR:** 0 **PR:** 8 **SD:** 46 **PD:** 31 ND 15	19 SAE TR	(98)

The bold text in the tables indicates subtitles and highlights randomized trials.

#### Reviewed trials

Early randomized trials, led by Dr. Theleen (PEMBRO-RT trial) (88) and Dr. Welsh (MDACC trial) in 2015 (89), ventured into this promising approach combining pembrolizumab with different radiotherapy regimens, but both faced challenges in patient stratification due to multiple confounding factors, complicating comparisons between treatment arms. The recruitment of patients without a PD-L1 status and the lack of randomization based on that key biomarker limits the interpretation of these two trials. For instance, patients with high PD-L1 expression were more frequent in the experimental arms (PEMBRO-RT 28% vs 13%; MDACC 35% vs 15%).

The Pembro-RT trial (NCT, phase, n=36 + 40) (88) compared pembrolizumab either alone or after the completion of SBRT (3 fractions of 8 Gy) to one lesion out of a median 3.5 lesions per patient. All patients had received previous chemotherapy, 42.1% had previous radiotherapy, but none had previous immunotherapy. The trial yielded a significant improvement in the out-of-flied DCR at 12 weeks (64% vs 40%; p = 0.042). The increased out-of-field ORR (36% vs 18%; p = 0.07 at 12 weeks), also referred to as systemic or abscopal response rate (ARR), didn’t meet the prespecified value of 50% as primary end point criteria for meaningful clinical benefit. The improvements of the median PFS (6.6 vs 1.9, HR 0.71, p =0.19) and OS (7.6 vs 15.9, HR 0.66, p = 0.16) were not significant either. The synergy was evidenced by an increased infiltration of cytotoxic T cells after SBRT (fold difference in CD103+ cytotoxic T-cells after 6 weeks: 4.87 vs 1.85). Subgroup analysis suggested that PD-L1-negative patients were the only ones to benefit from the combination therapy (ARR; 4% vs 22%, HR = 0.49, p = 0.14; PFS HR = 0.79, p = 0.03). These results suggest that SBRT may enhance the efficacy of pembrolizumab in patients with PD-L1-negative tumors, a population that typically responds poorly to immune checkpoint inhibitor (ICI) monotherapy.

At MD Anderson (NCT02444741, phase 1/2, n=72) (89), patients received either conventionally fractionated RT (CFRT 45 Gy in 15 fractions) or SBRT (50 Gy in 4 fractions) to 1 (90%) or 2 (10%) lesions, out of a median number of metastases at baseline of 3 (range 1–10). Patients were either newly diagnosed or previously treated, with the majority being ICI-naive. The control arm of the study consisted of pembrolizumab monotherapy. Salvage RT was allowed after progression in the control arm. The CFRT group demonstrated poorer baseline conditions and outcomes, including a marked reduction in absolute lymphocyte counts (ALC), which likely contributed to the lack of improvement in the overall population abscopal response rate (ARR) (25% vs 22%, p=0.992). This was further compounded by the fact that CFRT is more detrimental with respect to RT-induced lymphopenia. SBRT, on the other hand, demonstrated improved ARR results (38% vs 10%). This difference between the two RT regimens can be attributed to the fragility of patients unamenable for SBRT, the lower dose of conventional RT, and the mean reduction in ALC (19% with SBRT vs 47% with traditional RT; p=0.003). PFS was not significantly improved in the RT group (9.1 vs 5.1 months, p=0.520). Patients in the control arm who had previous RT had a better ARR (43% vs 20%; p=0.330). The PD-L1 subgroup analysis revealed inconsistent patterns. High expressors (experimental vs control arm) had similar ARR (25% vs 22%; p=0.992) but lower median PFS (5.6 vs 20.6; p=0.490). Low expressors had a better ARR (33% vs 0%; p=0.24) and significantly higher median PFS (20.8 vs 4.6 months; p=0.001). Of note, ARR (11% vs 30%) and PFS (7.8 vs 14.2; p=0.25) were in favor of pembrolizumab alone in the PD-L1 negative patients. These unexpected findings have no mechanistic rationale and are inherently inconclusive, as the study was neither designed nor powered for such subgroup comparisons. Future studies specifically designed and stratified by PD-L1 expression are required to determine which patients derive the most benefit.

Efforts have been made by the investigators of the PEMBRO-RT and MD Anderson trials to combine results in a pooled analysis, allowing a larger sample size (n=148) (89). Indeed, statistical significance was met in the improvement of ARR (41.7% vs 19.7%; p=0.0039), abscopal control rate (65.3% vs 43.4%; p=0.0071), median PFS (9.0 vs 4.4 months; p=0.045) and OS (19.2 vs 8.7 months; p=0.0004). The analysis also confirmed the superiority of SBRT over conventional RT. However, a pooled analysis cannot mitigate the limitations inherent to each trial. More bias can be introduced, particularly by creating a more heterogeneous population with variable treatment schedules and doses. For instance, the possible treatment opportunity in PD-L1 negative patients reported in the PEMBRO-RT trial was not sustained in the pooled analysis.

A smaller phase 1 trial (NCT03035890) (90) combined SBRT and standard of care ICI with (n=16) or without (n=19) concurrent chemotherapy. Patients were also offered a second course of RT (n=15) in case of progression. No G3+ radiation-induced toxicities were observed. The trial showed encouraging results with an ARR of 52.9%, a DCR of 73.5%, a median OS of 15 months, and a PFS of 6.9 months. Patients with high PD-L1 expression had an ARR of 64.3%. Notably, the few patients (3/15) who responded to re-RT after progression support the potential of RT to overcome ICI secondary resistance in a specific, but not yet defined, population.

Patients with highly metastatic tumors (median 10 metastases), mostly PD-L1 negative (71.4%) in the Combinatory ImmunoTherapy-1 trial (NCT03644823, phase 2, n=21) (91) didn’t demonstrate a significant benefit with 3 fractions of 6 Gy radiotherapy added to atezolizumab (ARR 19%, DCR 57.1%, median PFS 4.3 months). The investigators proposed that higher RT doses might yield better results.

Detecting meaningful differences in very advanced disease, characterized by widespread metastases and poor performance status, remains a significant challenge.

SBRT (27 to 30 Gy) initially appeared as a potential solution to enhance the limited efficacy of CTLA-4 monotherapy. While the NCT02221739 (phase 2, n=39) trial (92) didn’t yield strong results (ARR 17.9%, median OS 7.4 months, median PFS 3.8 months), it did provide insight into the abscopal effect, as a responding patient demonstrated CD8 T cell expansion targeting radiation-induced mutations and increased serum interferon-β, which are both indicators of an enhanced anti-tumor immune response.

Low-dose radiotherapy (LDRT) is a therapeutic approach that harnesses the immunomodulatory properties of radiation, with the potential to convert the tumor microenvironment (TME) into an immune-infiltrated, ‘hot’ phenotype, thereby enhancing susceptibility to the abscopal effect and the synergy with ICI. Based on this theory, the IHC trial (NCT03812549, phase 1, n=29) (93) combined LDRT (4 Gy in 2 fractions) to large tumors, with SBRT (30 Gy in 3 fractions) to smaller ones, and sintilimab, for PD-L1 positive patients. The trial resulted in encouraging ORR (56.3%) and median PFS (9 months). A more detailed analysis of this trial is awaited.

Multisite irradiation (1 to 6 sites), tailored to metastasis location (30 to 50 Gy), was also evaluated in the COSINR trial (NCT03223155, phase 1) (94). Tumors larger than 65 cm³ were partially irradiated. The trial showed promising results (ORR 45.9%, DCR 62.1%, ARR 33.3%, median PFS 5.8 months). This study also introduced the combination of nivolumab, ipilimumab, and SBRT with acceptable toxicity. These results appear to be slightly higher than the CheckMate 227 trial (ORR 35.9%, DCR 65.2%, median PFS 5.1 months) (95, 96) that led to the approval of the dual ICI in PD-L1 positive patients. Additionally, the trial explored the advantages of concurrent (n=18) versus sequential (n=19) regimens. Even though a direct comparison wasn’t feasible, the concurrent approach was selected for multi-institutional trial expansion, due to a better toxicity profile, longer PFS (7.9 vs 4.7), and comparable ORR. Preliminary analysis showed encouraging outcomes (ORR 54.2%, median PFS 5.7 months, OS 34 months) in a widely metastatic population (n=75), including a significant number of PD-L1 negative patients (46.7%). Results were even more encouraging in the PD-L1 positive subgroup (ORR 67.5% vs 36.7%; PFS 11 vs 4.1 months; OS 38 vs 16 months).

The tri-modality approach was also investigated in two other trials that were terminated. The first report of the NCT03168464 trial (phase1, n=9) (97) showed no G3+ TRAE, an ORR (22%) below the objective of 50%, and was terminated due to slow accrual. NCT02888743 (phase 2, n=78) (98) targeted third line PD-L1 refractory patients, a population with limited treatment options. The experimental arm consisted of durvalumab and tremelimumab with hyper-fractionated LDRT (8 Gy in 4 cycles of 0.5 Gy twice daily over 2 days) or 24 Gy in 3 fractions. The trial was terminated for futility due to the lack of significant improvement with the addition of RT to dual-ICI in neither ORR, PFS or OS. The investigators observed a decrease in systemic lymphocyte count, associated with disease progression, and possibly reflecting an immunosuppressive effect of radiotherapy. Despite the limited efficacy, these efforts demonstrated the safety of this combination, thereby enabling further exploration.

## The inherent gaps in clinical trials

Despite a remarkable 278% increase in the number of clinical trials investigating PD-1/PD-L1 inhibitors between 2016 and 2021, our review of the current clinical landscape highlights a persistent lack of statistical robustness across many studies. Trials evaluating the combination of RT and ICI are predominantly early-phase (I or II), with findings that often remain exploratory due to small cohort sizes and limited patient accrual.

Of the 322 trials identified through ClinicalTrials.gov, 39 (12%) have been suspended, withdrawn, or terminated—most commonly due to insufficient recruitment. Only 14% (45 trials) are randomized, and 22% (72 trials) have remained open and actively recruiting for over two years. These trends underscore systemic limitations in trial execution and design.

Key barriers to successful clinical trial implementation include patient recruitment difficulties, escalating study complexity, regulatory burdens, a shortage of qualified personnel, and the rapid evolution of scientific innovation. The rising cost of clinical trials is a major concern, as it significantly contributes to the overall financial burden of drug development (99, 100).

The intense pace of advancement in immuno-oncology has also contributed to heightened competition among investigational therapies, resulting in the premature discontinuation of numerous trials once the clinical niche they sought to fill has been addressed by parallel developments. While this reflects the dynamism of the field, it also underscores the urgency of optimizing clinical development strategies to maximize resource efficiency and accelerate patient access to effective therapies.

Moreover, ethical considerations impose necessary but strict constraints on trial design. Experimental interventions must be grounded in strong scientific rationale, demonstrate an acceptable toxicity profile, and must not deprive patients of therapies known to be superior. These requirements may limit the feasibility of testing certain combinations, escalating doses, or isolating the effects of specific components.

In this context, innovative computational approaches have emerged as a powerful tool to integrate mechanistic insights with clinical data, refining treatment strategies in silico before clinical evaluation.

## Virtual clinical trials

### A novel complement to bridge gaps in traditional research

The fundamental constraints of conventional clinical trials are largely mitigated when utilizing virtual patient populations. Virtual clinical trials enable unrestricted flexibility in protocol design and patient cohort selection while offering an unlimited sample size, thereby ensuring near-optimal statistical power. Moreover, common biases inherent to traditional clinical research—such as selection, randomization, and measurement biases—are minimized in virtual studies. These trials can be effectively conducted through quantitative systems pharmacology (QSP) modeling, providing a robust framework for simulating complex biological and therapeutic interactions. This discipline is a multidisciplinary approach that combines insights from biomedical sciences, mathematical modeling, and computational techniques to simulate the behavior of biological systems and pharmacological dynamics.

Initially introduced in preclinical research, QSP has progressively emerged as a pivotal tool in the design and analysis of clinical trials, particularly in oncology. The increasing regulatory recognition of this approach is reflected by the exponential rise in QSP-based submissions to the FDA, which reportedly double approximately every 1.4 years (101). This approach enables the early prediction of treatment efficacy and toxicity, providing crucial quantitative insights into dynamic biological and pharmacological interactions. By refining therapeutic strategies, QSP facilitates the identification of patient subpopulations most likely to respond to treatment, thereby optimizing clinical trial design. Moreover, QSP-driven virtual trials contribute to better understanding biological mechanisms, discovering new biomarkers, limiting the risk of trial failure, reducing development costs, and accelerating the clinical translation of novel therapeutics. The integration of these predictive models into clinical research not only enhances decision-making but also represents a transformative step toward the advancement of precision oncology.

### The case of the QSP-IO platform

A prominent example of this modeling strategy is the QSP-IO platform, developed by Richard J. Sové et al. (102) to provide a foundational framework to simulate the core components of the tumor-immune dynamics in NSCLC, alongside the pharmacokinetics and pharmacodynamics of ICIs. This toolbox is modular by design, allowing for the integration of additional components in future, goal-oriented simulations. This platform was subsequently extended within our team by Miriam Schirru and co-authors (103), by integrating the immunomodulatory effects of radiotherapy, and enabling the translation of simulated tumor dynamics into clinical outcomes based on RECIST criteria.

More precisely, the QSP model recapitulates the tumor immune cycle as conceptualized by Mellman et al. (104), partitioned into four interconnected compartments: the tumor, the tumor-draining lymph node, the central (blood) compartment, and the lymphatic transport system. Of particular relevance to the study of the RT-ICI synergy, the model quantifies the RT-induced immunogenic tumor cell death, thereby generating tumor-associated antigens that are captured by antigen-presenting cells. This initiates a cascade of immunological events culminating in the activation and infiltration of effector T cells into the tumor microenvironment. In the presence of ICIs, these T cells can effectively mediate tumor cell killing and propagate the cycle further.

This QSP enriched model has been calibrated and validated against multiple clinical datasets, including the clinical trial by Ye et al. (105) as incorporated in the work of M. Schirru et al. (103), and the PEMBRO-RT trial, as presented by H. Charef et al. (106). The model was able to reproduce observed clinical response rates with minimal to no deviation and is currently under further analysis to explore the broader dynamics of anti-tumor immunity.

In the context of investigating the RT-ICI synergy, the model’s ability to predict treatment outcomes was leveraged to compare various therapeutic scenarios. Six distinct protocols were simulated to explore the optimal dosing and scheduling strategies, comparing nivolumab monotherapy with sequential and concurrent combinations using different RT regimens. The simulations also shed light on the impact of RT alone in advanced disease, an intervention not ethically feasible to assess in the clinical practice, yet offering valuable mechanistic insights.

Notably, the addition of RT to nivolumab significantly improved the ORR at day 400, from 21% with nivolumab alone, reaching up to 56%, which exceeds the additive effect of each treatment alone, and thereby confirming the synergistic potential of this approach.

Furthermore, the concurrent combination of RT and nivolumab substantially outperformed the sequential administration, yielding ORRs of 56% and 26%, respectively. The authors suggest that delays between treatment modalities may allow tumor regrowth or resistance, ultimately hindering long-term efficacy.

In the sequential setting, a single dose of RT (30 Gy) yielded a superior ORR (37%) compared to hypofractionated RT (HFRT; 3 × 8 Gy, ORR 26%). In the concomitant setting, conventional RT (60 Gy over 6 weeks) and HFRT achieved comparable ORRs (51% and 56%, respectively), while the latter yielding more patients responding beyond the duration of the trials (195 vs 133 censored patients in the duration of response evaluation).

Further investigation is warranted to refine the comparative performance of these RT regimens and combination sequencing. Ongoing work from our pharmacometrics team at the University of Montréal aims to enhance the platform’s ability to more realistically mirror the intricacies of tumor and immune dynamics following radiotherapy, improve its external validity through advanced virtual population generation techniques, and employ innovative dynamical systems analysis to predict the trajectory of a tumor toward a “basin of attraction”, leading to either a tumor-free state or maximal tumor volume (107). The latter can also be used to determine the earliest time at which the therapy can be stopped, reducing drug exposure without jeopardizing its efficacy (108).

## Conclusion

NSCLC remains a primary focus of research due to its high prevalence and mortality rates. The revolutionary journey of ICIs has rapidly reshaped the landscape of cancer therapy and is poised to continue unfolding new frontiers. Radiotherapy has been a cornerstone of cancer therapy for over a century and continues to thrive with groundbreaking discoveries that drive the development of highly precise irradiation techniques.

The potential synergy between RT and ICIs, along with discussions around the clinical relevance of the abscopal effect, divides the scientific community. RT has complex metabolic, vascular, and immune effects, both locally and systemically. The observed double-edged effects of RT underscore the critical need to deepen our understanding of immune dynamics. The identification of more reliable biomarkers, such as TIM3+ (109), can better inform and predict the outcome of the combination.

In resectable tumors, RT is arguably the treatment most capable of enhancing pathological response and providing the immune activation necessary for ICI activity. With the limitations surrounding pCR as a predictive outcome, future trials should aim to confirm the superiority of ICI-RT over ICI-CT through long-term outcome data. Based on the available evidence, albeit from studies with small sample sizes, the concerns regarding surgery delays and complications are lessened. While a combination of RT, CT, and ICI may be feasible for N2 tumors, the proven efficacy of ICI monotherapy in advanced stages makes the quadri-modality approach less justifiable, particularly for PD-L1 positive patients. Depending on the clinical context, high-dose radiotherapy may not be warranted for non-ablative purposes. Conversely, the benefit of ICI - 54 Gy RT in inoperable early stages is clearly demonstrated in the reviewed trials. The results of ongoing phase III trials are eagerly awaited to enable the clinical adoption of this combination.

LA-NSCLC includes a wide range of subcategories, marked by a high variability in clinical outcomes. The approval of consolidation durvalumab after CRT is a turning point that served as a platform for further research. Evidence suggests that early ICI integration may yield better outcomes. Despite an uncertain risk-benefit ratio, the synergistic potential and the large possible impact justify the ongoing investigations.

Conventional radiotherapy remains the standard of care at this stage. However, the incorporation of SBRT is likely to become increasingly prominent in the future. The trials examined in this paper highlight the building momentum behind personalized RT, customized based on tumor size and nodal involvement, with the prospect of adjustments throughout the treatment process. Within this innovative framework, a transition away from chemotherapy could be conceivable.

Advanced stages represent the most relevant setting to illustrate the abscopal effect through the out-of-field response to ICI-RT. However, significant differences remain difficult to identify, which underscores the need to adequately design future trials by targeting the subpopulations most likely to benefit. Re-irradiation after progression seems feasible and, in theory, could help overcome secondary resistance to ICI. Other promising individualized approaches include multisite or partial irradiation of large tumors. Additionally, combining dual ICI with RT demonstrates manageable toxicity, offering new possibilities for further investigation.

Whether these results stem from a mechanistic synergy and the abscopal effect, or rather a strategic integration of localized and systemic treatments, the ICI-RT combination holds considerable promise for enhancing patient outcomes.

Combined treatments extend beyond ICIs to include conventional (e.g., chemotherapy, targeted therapy, angiogenesis modulators) and unconventional strategies (e.g., local anesthetics, traditional Chinese medicine), as well as emerging approaches, such as oncolytic viruses, metabolic and gut microbiota modulators. The complex mechanistic rationale underlying combined treatments justifies placing them at the forefront of cancer research, with the aim of enhancing therapeutic efficacy, overcoming resistance, and paving the way for multimodal, individualized treatment protocols. The abundance of innovation and the large range of clinical contexts creates a competitive race to market that demands meticulous planning. In silico predictions can play a crucial role, whether through virtual trials to identify optimal treatment scenarios or patient “twin” models to personalize treatment strategies and aid clinical decision-making.

The modeling domain is often constrained by limited data availability. This review offers a mechanistic and clinical understanding of the combination of RT-ICI, highlights the most relevant research opportunities, and provides a dataset used to support the validation and refinement of predictive computational models.

## Glossary

3D-CRT: 3D Conformal Radiation Therapy
AE: Adverse events
ALC: Absolute lymphocyte counts
ALK: Anaplastic Lymphoma Kinase
APC: Antigen presenting cells
ARR: Abscopal response rate
ATP: Adenosine triphosphate
BRAF: B-Raf Proto-Oncogene
c-CRT: Concurrent Chemoradiotherapy
CFRT: Conventionally fractionated Radiation Therapy
CR: Complete response
CRT: Chemoradiotherapy
CT: Chemotherapy
CTL: Cytotoxic lymphocyte
CTLA-4: Cytotoxic T-lymphocyte associated protein 4
CTRL: Control
DAMP: Damage-associated molecular patterns
DC: Dendritic cell
DCR: Disease control rate
DNA: Deoxyribonucleic acid
ECOG: Eastern Cooperative Oncology Group
EGFR: Epidermal growth factor receptor
EXP: Experimental
FDA: Food and Drug Administration
Fx: Fraction
G3+: Grade 3 or higher
Gy: Gray
HIF-1α: Hypoxia-inducible factor 1-α
HMGB1: High mobility group protein 1
HSP: Heat shock proteins
ICI: Immune checkpoint inhibitors
IFN: Interferons
IGRT: Image Guided Radiation Therapy
IL-6: Interleukin-6
IMRT: Intensity Modulated Radiation Therapy
irAE: Immune-related adverse event
LA-NSCLC: Locally Advanced NSCLC
LAG-3: Lymphocyte-activation gene 3
LDRT: Low dose radiotherapy
MDACC: MD Anderson Cancer Center
MDSC: Myeloid derived suppressor cells
MHC: Major histocompatibility complex
MPR: Major pathological response
NCCN: National Comprehensive Cancer Network
NK: Natural killers
NLM: United States National Library of Medicine
NSCLC: Non-small-cell lung cancer
ORR: Overall response rate
OS: Overall survival
pCR: Pathological complete response
PD-1: Programmed cell death protein 1
PD-L1: Programmed death-ligand 1
PFS: Progression free survival
RIL: Radiotherapy-induced lymphopenia
RLI: Radiotherapy-induced lymphopenia
ROS1: ROS proto-oncogene 1
RT: Radiotherapy
SBRT: Stereotactic ablative radiotherapy
SBRT: Stereotactic Body Radiation Therapy
SD: Stable disease
Squam. histol.: Squamous histology
TAM: Tumor-associated macrophage
TCR: T cell receptor
TGF-β: Transforming growth factor-β
TIL: Tumor-infiltrating lymphocyte
Tim3: T-cell immunoglobulin and mucin-domain containing protein 3
TME: Tumor microenvironment
TNF-α: Tumor necrosis factor-α
TPS: Tumor Proportion Score
Treg: Regulatory T cells
Resource Identification Initiative: ClinicalTrials.gov (RRID: SCR_002309).

## References

[1] Sung H Ferlay J Siegel RL Laversanne M Soerjomataram I Jemal A . Global cancer statistics 2020: GLOBOCAN estimates of incidence and mortality worldwide for 36 cancers in 185 countries. CA A Cancer J Clin. (2021) 71:209–49. doi: 10.3322/caac.21660, PMID: 33538338

[2] SEER . Explorer: An interactive website for SEER cancer statistics. In: Surveillance research program, national cancer institute Bethesda, MD: National Cancer Institute (2024). Available online at: https://seer.cancer.gov/explorer/ (Accessed June 10, 2024).

[3] Brenner DR Poirier A Woods RR Ellison LF Billette JM Demers AA . Projected estimates of cancer in Canada in 2022. CMAJ. (2022) 194:E601–7. doi: 10.1503/cmaj.212097, PMID: 35500919 PMC9067380

[4] Sonkin D Thomas A Teicher BA . Cancer treatments: Past, present, and future. Cancer Genet. (2024) 286–287:18–24. doi: 10.1016/j.cancergen.2024.06.002, PMID: 38909530 PMC11338712

[5] Hanahan D Weinberg RA . Hallmarks of cancer: the next generation. Cell. (2011) 144:646–74. doi: 10.1016/j.cell.2011.02.013, PMID: 21376230

[6] Edvard Smith CI Holmdahl R Kämpe O Kärre K . Scientific Background: Discovery of cancer therapy by inhibition of negative immune regulation (2018). Karolinska Institutet. Available online at: https://www.nobelprize.org/uploads/2018/10/advanced-medicineprize2018.pdf (Accessed June 29, 2024).

[7] Rückert M Flohr AS Hecht M Gaipl US . Radiotherapy and the immune system: More than just immune suppression. Stem Cells. (2021) 39:1155–65. doi: 10.1002/stem.3391, PMID: 33961721

[8] Lisi L Lacal PM Martire M Navarra P Graziani G . Clinical experience with CTLA-4 blockade for cancer immunotherapy: From the monospecific monoclonal antibody ipilimumab to probodies and bispecific molecules targeting the tumor microenvironment. Pharmacol Res. (2022) 175:105997. doi: 10.1016/j.phrs.2021.105997, PMID: 34826600

[9] Morad G Helmink BA Sharma P Wargo JA . Hallmarks of response, resistance, and toxicity to immune checkpoint blockade. Cell. (2021) 184:5309–37. doi: 10.1016/j.cell.2021.09.020, PMID: 34624224 PMC8767569

[10] Doki Y Ajani JA Kato K Xu J Wyrwicz L Motoyama S . Nivolumab combination therapy in advanced esophageal squamous-cell carcinoma. N Engl J Med. (2022) 386:449–62. doi: 10.1056/NEJMoa2111380, PMID: 35108470

[11] Olivares-Hernández A González Del Portillo E Tamayo-Velasco Á Figuero-Pérez L Zhilina-Zhilina S Fonseca-Sánchez E . Immune checkpoint inhibitors in non-small cell lung cancer: from current perspectives to future treatments—a systematic review. Ann Transl Med. (2023) 11:354–4. doi: 10.21037/atm-22-4218, PMID: 37675322 PMC10477621

[12] Behrouzieh S Sheida F Rezaei N . Review of the recent clinical trials for PD-1/PD-L1 based lung cancer immunotherapy. Expert Rev Anticancer Ther. (2021) 21:1355–70. doi: 10.1080/14737140.2021.1996230, PMID: 34686070

[13] Oyewole-Said D Konduri V Vazquez-Perez J Weldon SA Levitt JM Decker WK . Beyond T-cells: functional characterization of CTLA-4 expression in immune and non-immune cell types. Front Immunol. (2020) 11:608024. doi: 10.3389/fimmu.2020.608024, PMID: 33384695 PMC7770141

[14] Hossen MM Ma Y Yin Z Xia Y Du J Huang JY . Current understanding of CTLA-4: from mechanism to autoimmune diseases. Front Immunol. (2023) 14:1198365. doi: 10.3389/fimmu.2023.1198365, PMID: 37497212 PMC10367421

[15] Xu H Tan P Zheng X Huang Y Lin T Wei Q . Immune-related adverse events following administration of anti-cytotoxic T-lymphocyte-associated protein-4 drugs: a comprehensive systematic review and meta-analysis. DDDT. (2019) 13:2215–34. doi: 10.2147/DDDT.S196316, PMID: 31308633 PMC6613615

[16] Choi J Lee SY . Clinical characteristics and treatment of immune-related adverse events of immune checkpoint inhibitors. Immune Netw. (2020) 20:e9. doi: 10.4110/in.2020.20.e9, PMID: 32158597 PMC7049586

[17] Zhang R Cai XL Liu L Han XY Ji LN . Type 1 diabetes induced by immune checkpoint inhibitors. Chin Med J. (2020) 133:2595–8. doi: 10.1097/CM9.0000000000000972, PMID: 32842016 PMC7722553

[18] Hodi FS Chiarion-Sileni V Gonzalez R Grob JJ Rutkowski P Cowey CL . Nivolumab plus ipilimumab or nivolumab alone versus ipilimumab alone in advanced melanoma (CheckMate 067): 4-year outcomes of a multicentre, randomised, phase 3 trial. Lancet Oncol. (2018) 19:1480–92. doi: 10.1016/S1470-2045(18)30700-9, PMID: 30361170

[19] Zhang Z Liu X Chen D Yu J . Radiotherapy combined with immunotherapy: the dawn of cancer treatment. Sig Transduct Target Ther. (2022) 7:258. doi: 10.1038/s41392-022-01102-y, PMID: 35906199 PMC9338328

[20] De Andrade Carvalho H Villar RC . Radiotherapy and immune response: the systemic effects of a local treatment. Clinics. (2018) 73:e557s. doi: 10.6061/clinics/2018/e557s, PMID: 30540123 PMC6257057

[21] Barker HE Paget JTE Khan AA Harrington KJ . The tumour microenvironment after radiotherapy: mechanisms of resistance and recurrence. Nat Rev Cancer. (2015) 15:409–25. doi: 10.1038/nrc3958, PMID: 26105538 PMC4896389

[22] Campian JL Ye X Brock M Grossman SA . Treatment-related lymphopenia in patients with stage III non-small-cell lung cancer. Cancer Invest. (2013) 31:183–8. doi: 10.3109/07357907.2013.767342, PMID: 23432821 PMC4596242

[23] Upadhyay R Venkatesulu BP Giridhar P Kim BK Sharma A Elghazawy H . Risk and impact of radiation related lymphopenia in lung cancer: A systematic review and meta-analysis. Radiotherapy Oncol. (2021) 157:225–33. doi: 10.1016/j.radonc.2021.01.034, PMID: 33577865

[24] Deek MP Benenati B Kim S Chen T Ahmed I Zou W . Thoracic vertebral body irradiation contributes to acute hematologic toxicity during chemoradiation therapy for non-small cell lung cancer. Int J Radiat OncologyBiologyPhysics. (2016) 94:147–54. doi: 10.1016/j.ijrobp.2015.09.022, PMID: 26700708 PMC5767469

[25] Yankelevitz DF Henschke CI Knapp PH Nisce L Yi Y Cahill P . Effect of radiation therapy on thoracic and lumbar bone marrow: evaluation with MR imaging. Am J Roentgenology. (1991) 157:87–92. doi: 10.2214/ajr.157.1.1904679, PMID: 1904679

[26] Kapoor V Collins A Griffith K Ghosh S Wong N Wang X . Radiation induces iatrogenic immunosuppression by indirectly affecting hematopoiesis in bone marrow. Oncotarget. (2020) 11:1681–90. doi: 10.18632/oncotarget.27564, PMID: 32477458 PMC7233802

[27] Li Y Fan X Pei Y Wu K . Dynamic effects of thoracic irradiation on immune status of organs in and out of radiation field in mice. Int J Radiat OncologyBiologyPhysics. (2023) 117:e244. doi: 10.1016/j.ijrobp.2023.06.1177

[28] Paganetti H . A review on lymphocyte radiosensitivity and its impact on radiotherapy. Front Oncol. (2023) 13:1201500. doi: 10.3389/fonc.2023.1201500, PMID: 37601664 PMC10435323

[29] Ellsworth SG . Field size effects on the risk and severity of treatment-induced lymphopenia in patients undergoing radiation therapy for solid tumors. Adv Radiat Oncol. (2018) 3:512–9. doi: 10.1016/j.adro.2018.08.014, PMID: 30370350 PMC6200885

[30] Abravan A Faivre-Finn C Kennedy J McWilliam A Van Herk M . Radiotherapy-related lymphopenia affects overall survival in patients with lung cancer. J Thorac Oncol. (2020) 15:1624–35. doi: 10.1016/j.jtho.2020.06.008, PMID: 32553694

[31] Xia WY Feng W Zhang CC Shen YJ Zhang Q Yu W . Radiotherapy for non-small cell lung cancer in the immunotherapy era: the opportunity and challenge—a narrative review. Transl Lung Cancer Res. (2020) 9:2120–36. doi: 10.21037/tlcr-20-827, PMID: 33209631 PMC7653139

[32] Rückert M Deloch L Frey B Schlücker E Fietkau R Gaipl US . Combinations of radiotherapy with vaccination and immune checkpoint inhibition differently affect primary and abscopal tumor growth and the tumor microenvironment. Cancers. (2021) 13:714. doi: 10.3390/cancers13040714, PMID: 33572437 PMC7916259

[33] Pangal DJ Yarovinsky B Cardinal T Cote DJ Ruzevick J Attenello FJ . The abscopal effect: systematic review in patients with brain and spine metastases. Neuro-Oncology Adv. (2022) 4:vdac132. doi: 10.1093/noajnl/vdac132, PMID: 36199973 PMC9529003

[34] Mole RH . Whole body irradiation—Radiobiology or medicine? Br J Radiol. (1953) 26:234–41. doi: 10.1259/0007-1285-26-305-234, PMID: 13042090

[35] Abuodeh Y Venkat P Kim S . Systematic review of case reports on the abscopal effect. Curr Problems Cancer. (2016) 40:25–37. doi: 10.1016/j.currproblcancer.2015.10.001, PMID: 26582738

[36] Demaria S Formenti SC . The abscopal effect 67 years later: from a side story to center stage. Br J Radiol. (2020) 93:20200042. doi: 10.1259/bjr.20200042, PMID: 32101479 PMC7217574

[37] Dagoglu N Karaman S Caglar HB Oral EN . Abscopal effect of radiotherapy in the immunotherapy era: systematic review of reported cases. Cureus. (2019) 11:e4103. doi: 10.7759/cureus.4103, PMID: 31057997 PMC6476623

[38] Gao W Wang X Zhou Y Wang X Yu Y . Autophagy, ferroptosis, pyroptosis, and necroptosis in tumor immunotherapy. Sig Transduct Target Ther. (2022) 7:196. doi: 10.1038/s41392-022-01046-3, PMID: 35725836 PMC9208265

[39] Liu S Wang W Hu S Jia B Tuo B Sun H . Radiotherapy remodels the tumor microenvironment for enhancing immunotherapeutic sensitivity. Cell Death Dis. (2023) 14:679. doi: 10.1038/s41419-023-06211-2, PMID: 37833255 PMC10575861

[40] Ost D Goldberg J Rolnitzky L Rom WN . Survival after surgery in stage IA and IB non–small cell lung cancer. Am J Respir Crit Care Med. (2008) 177:516–23. doi: 10.1164/rccm.200706-815OC, PMID: 18006887 PMC2258444

[41] Deutsch JS Cimino-Mathews A Thompson E Provencio M Forde PM Spicer J . Association between pathologic response and survival after neoadjuvant therapy in lung cancer. Nat Med. (2024) 30:218–28. doi: 10.1038/s41591-023-02660-6, PMID: 37903504 PMC10803255

[42] Tan VS Correa RJM Nguyen TK Louie AV Malthaner RA Fortin D . Measuring the integration of stereotactic ablative radiotherapy plus surgery for early-stage non-small cell lung cancer: long-term clinical outcomes. Int J Radiat OncologyBiologyPhysics. (2025) 121:39–44. doi: 10.1016/j.ijrobp.2024.07.2332, PMID: 39362312

[43] Wakelee H Liberman M Kato T Tsuboi M Lee SH Gao S . Perioperative pembrolizumab for early-stage non–small-cell lung cancer. N Engl J Med. (2023) 389:491–503. doi: 10.1056/NEJMoa2302983, PMID: 37272513 PMC11074923

[44] Cascone T Awad MM Spicer JD He J Lu S Sepesi B . Perioperative nivolumab in resectable lung cancer. N Engl J Med. (2024) 390:1756–69. doi: 10.1056/NEJMoa2311926, PMID: 38749033

[45] Schvartsman G Rezende ACPD Gomes DBD Koch LDO Bibas BJ Gomes O . Stereotactic ablative radiotherapy with nivolumab for early-stage operable non-small cell lung cancer: A phase 2 study. JCO. (2023) 41:8554–4. doi: 10.1200/JCO.2023.41.16_suppl.8554

[46] Schvartsman G . Stereotactic ablative radiotherapy in combination with nivolumab for early stage operable non-small cell lung cancer: A phase II study (2024). BARCELONA. Available online at: https://cslide.ctimeetingtech.com/esmo2024/attendee/confcal_2/presentation (Accessed October 21, 2024).

[47] Lee B Mynard N Nasar A Villena-Vargas J Chow O Harrison S . Surgical resection after neoadjuvant durvalumab and radiation is feasible and safe in non–small cell lung cancer: Results from a randomized trial. J Thorac Cardiovasc Surgery. (2023) 165:327–34. doi: 10.1016/j.jtcvs.2022.07.017, PMID: 36028357

[48] Altorki NK McGraw TE Borczuk AC Saxena A Port JL Stiles BM . Neoadjuvant durvalumab with or without stereotactic body radiotherapy in patients with early-stage non-small-cell lung cancer: a single-centre, randomised phase 2 trial. Lancet Oncol. (2021) 22:824–35. doi: 10.1016/S1470-2045(21)00149-2, PMID: 34015311

[49] Altorki NK Walsh ZH Melms JC Port JL Lee BE Nasar A . Neoadjuvant durvalumab plus radiation versus durvalumab alone in stages I–III non-small cell lung cancer: survival outcomes and molecular correlates of a randomized phase II trial. Nat Commun. (2023) 14:8435. doi: 10.1038/s41467-023-44195-x, PMID: 38114518 PMC10730562

[50] Albain KS Swann RS Rusch VW Turrisi AT Shepherd FA Smith C . Radiotherapy plus chemotherapy with or without surgical resection for stage III non-small-cell lung cancer: a phase III randomised controlled trial. Lancet. (2009) 374:379–86. doi: 10.1016/S0140-6736(09)60737-6, PMID: 19632716 PMC4407808

[51] Lee J Cho J Lee C Lee CY Lee JG Kim DJ . Phase Ib study of neoadjuvant concurrent chemoradiation plus durvalumab followed by surgery and adjuvant durvalumab for resectable stage III NSCLC. JCO. (2023) 41:8556–6. doi: 10.1200/JCO.2023.41.16_suppl.8556

[52] National Cancer Institute . Non-small cell lung cancer treatment (PDQ^®^)–Health professional version (2025). Bethesda, MD: National Cancer Institute. Available online at: https://www.cancer.gov/types/lung/hp/non-small-cell-lung-treatment-pdq (Accessed March 16, 2025).

[53] Wu TC Stube A Felix C Oseguera D Romero T Goldman J . Safety and efficacy results from iSABR, a phase 1 study of stereotactic ABlative radiotherapy in combination with durvalumab for early-stage medically inoperable non-small cell lung cancer. Int J Radiat OncologyBiologyPhysics. (2023) 117:118–22. doi: 10.1016/j.ijrobp.2023.03.069, PMID: 37023987

[54] Monjazeb AM Daly ME Luxardi G Maverakis E Merleev AA Marusina AI . Atezolizumab plus stereotactic ablative radiotherapy for medically inoperable patients with early-stage non-small cell lung cancer: a multi-institutional phase I trial. Nat Commun. (2023) 14:5332. doi: 10.1038/s41467-023-40813-w, PMID: 37658083 PMC10474145

[55] Chang JY Lin SH Dong W Liao Z Gandhi SJ Gay CM . Stereotactic ablative radiotherapy with or without immunotherapy for early-stage or isolated lung parenchymal recurrent node-negative non-small-cell lung cancer: an open-label, randomised, phase 2 trial. Lancet. (2023) 402:871–81. doi: 10.1016/S0140-6736(23)01384-3, PMID: 37478883 PMC10529504

[56] Robinson C Hu C Machtay M Newton M Wu K Barrett K . P1.18–12 PACIFIC-4/RTOG 3515: phase III study of durvalumab following SBRT for unresected stage I/II, lymph-node negative NSCLC. J Thorac Oncol. (2019) 14:S630–1. doi: 10.1016/j.jtho.2019.08.1328

[57] Jabbour SK Houghton B Robinson AG Quantin X Wehler T Kowalski D . Phase 3, randomized, placebo-controlled study of stereotactic body radiotherapy (SBRT) with or without pembrolizumab in patients with unresected stage I or II non–small cell lung cancer (NSCLC): KEYNOTE-867. JCO. (2022) 40:TPS8597–TPS8597. doi: 10.1200/JCO.2022.40.16_suppl.TPS8597

[58] Daly ME Redman M Simone CB Monjazeb AM Bauman JR Hesketh P . SWOG/NRG S1914: A randomized phase III trial of induction/consolidation atezolizumab + SBRT vs. SBRT alone in high risk, early-stage NSCLC (NCT04214262). Int J Radiat OncologyBiologyPhysics. (2022) 114:e414. doi: 10.1016/j.ijrobp.2022.07.1600

[59] Casal-Mouriño A Ruano-Ravina A Lorenzo-González M Rodríguez-Martínez Á Giraldo-Osorio A Varela-Lema L . Epidemiology of stage III lung cancer: frequency, diagnostic characteristics, and survival. Transl Lung Cancer Res. (2021) 10:506–18. doi: 10.21037/tlcr.2020.03.40, PMID: 33569332 PMC7867742

[60] Bradley JD Paulus R Komaki R Masters G Blumenschein G Schild S . Standard-dose versus high-dose conformal radiotherapy with concurrent and consolidation carboplatin plus paclitaxel with or without cetuximab for patients with stage IIIA or IIIB non-small-cell lung cancer (RTOG 0617): a randomised, two-by-two factorial phase 3 study. Lancet Oncol. (2015) 16:187–99. doi: 10.1016/S1470-2045(14)71207-0, PMID: 25601342 PMC4419359

[61] Yegya-Raman N Zou W Nie K Malhotra J Jabbour SK . Advanced radiation techniques for locally advanced non-small cell lung cancer: intensity-modulated radiation therapy and proton therapy. J Thorac Dis. (2018) 10:S2474–91. doi: 10.21037/jtd.2018.07.29, PMID: 30206493 PMC6123184

[62] Simone CB Hu C Heinzerling JH Mileham K Higgins KA Lin L . NRG LU008: phase III prospective randomized trial of primary lung tumor stereotactic body radiation therapy (SBRT) followed by concurrent mediastinal chemoradiation for locally-advanced non-small cell lung cancer (LA-NSCLC). Int J Radiat OncologyBiologyPhysics. (2024) 120:e63. doi: 10.1016/j.ijrobp.2024.07.1917

[63] Lee P Luterstein E Goldman J Garon E Lee JM Felix C . Accelerated hypofractionated CRT followed by SABR boost (HyCRT-SABR) for locally advanced unresectable NSCLC: A prospective phase II radiation dose-escalation study. Int J Radiat OncologyBiologyPhysics. (2019) 105:S44. doi: 10.1016/j.ijrobp.2019.06.469

[64] Antonia SJ Villegas A Daniel D Vicente D Murakami S Hui R . Durvalumab after chemoradiotherapy in stage III non–small-cell lung cancer. N Engl J Med. (2017) 377:1919–29. doi: 10.1056/NEJMoa1709937, PMID: 28885881

[65] Spigel DR Faivre-Finn C Gray JE Vicente D Planchard D Paz-Ares L . Five-year survival outcomes from the PACIFIC trial: durvalumab after chemoradiotherapy in stage III non–small-cell lung cancer. JCO. (2022) 40:1301–11. doi: 10.1200/JCO.21.01308, PMID: 35108059 PMC9015199

[66] Durm GA Jabbour SK Althouse SK Liu Z Sadiq AA Zon RT . A phase 2 trial of consolidation pembrolizumab following concurrent chemoradiation for patients with unresectable stage III non–small cell lung cancer: Hoosier Cancer Research Network LUN 14-179. Cancer. (2020) 126:4353–61. doi: 10.1002/cncr.33083, PMID: 32697352 PMC10865991

[67] Jabbour SK Berman AT Decker RH Lin Y Feigenberg SJ Gettinger SN . Phase 1 trial of pembrolizumab administered concurrently with chemoradiotherapy for locally advanced non–small cell lung cancer: A nonrandomized controlled trial. JAMA Oncol. (2020) 6:848. doi: 10.1001/jamaoncol.2019.6731, PMID: 32077891 PMC7042914

[68] Jabbour SK Lee KH Frost N Breder V Kowalski DM Pollock T . Pembrolizumab plus concurrent chemoradiation therapy in patients with unresectable, locally advanced, stage III non–small cell lung cancer: the phase 2 KEYNOTE-799 nonrandomized trial. JAMA Oncol. (2021) 7:1351. doi: 10.1001/jamaoncol.2021.2301, PMID: 34086039 PMC8446818

[69] Reck M Lee KH Frost N Breder VV Kowalski D Levchenko E . Two-year update from KEYNOTE-799: Pembrolizumab plus concurrent chemoradiation therapy (cCRT) for unresectable, locally advanced, stage III NSCLC. JCO. (2022) 40:8508–8. doi: 10.1200/JCO.2022.40.16_suppl.8508

[70] Reck M Lee KH Frost N Breder V Kowalski DM Levchenko E . 1860: Pembrolizumab (pembro) plus concurrent chemoradiation therapy (cCRT) in unresectable locally advanced non-small cell lung cancer (NSCLC): Final analysis of KEYNOTE-799. J Thorac Oncol. (2025) 20:S124–5. doi: 10.1016/S1556-0864(25)00380-6

[71] Liu Y Yao L Kalhor N Carter BW Altan M Blumenschein G . Final efficacy outcomes of atezolizumab with chemoradiation for unresectable NSCLC: The phase II DETERRED trial. Lung Cancer. (2022) 174:112–7. doi: 10.1016/j.lungcan.2022.10.006, PMID: 36371941

[72] Lin SH Lin Y Yao L Kalhor N Carter BW Altan M . Phase II trial of concurrent atezolizumab with chemoradiation for unresectable NSCLC. J Thorac Oncol. (2020) 15:248–57. doi: 10.1016/j.jtho.2019.10.024, PMID: 31778797

[73] Peters S Felip E Dafni U Tufman A Guckenberger M Álvarez R . Progression-free and overall survival for concurrent nivolumab with standard concurrent chemoradiotherapy in locally advanced stage IIIA-B NSCLC: results from the european thoracic oncology platform NICOLAS phase II trial (European thoracic oncology platform 6-14). J Thorac Oncol. (2021) 16:278–88. doi: 10.1016/j.jtho.2020.10.129, PMID: 33188912

[74] Ross HJ Kozono DE Urbanic JJ Williams TM DuFrane C Bara I . Phase II trial of neoadjuvant and adjuvant atezolizumab and chemoradiation (CRT) for stage III non-small cell lung cancer (NSCLC). JCO. (2021) 39:8513–3. doi: 10.1200/JCO.2021.39.15_suppl.8513

[75] Bradley JD Nishio M Okamoto I Newton MD Trani L Shire NJ . PACIFIC-2: Phase 3 study of concurrent durvalumab and platinum-based chemoradiotherapy in patients with unresectable, stage III NSCLC. JCO. (2019) 37:TPS8573–TPS8573. doi: 10.1200/JCO.2019.37.15_suppl.TPS8573

[76] Jabbour SK Cho BC Bria E Kato T Bhosle J Gainor JF . Rationale and design of the phase III KEYLYNK-012 study of pembrolizumab and concurrent chemoradiotherapy followed by pembrolizumab with or without olaparib for stage III non-small-cell lung cancer. Clin Lung Cancer. (2022) 23:e342–6. doi: 10.1016/j.cllc.2022.04.003, PMID: 35618629 PMC10865425

[77] Varlotto JM Sun Z Ramalingam SS Wakelee HA Lovly CM Oettel KR . Randomized phase III Trial of MEDI4736 (durvalumab) as concurrent and consolidative therapy or consolidative therapy alone for unresectable stage 3 NSCLC: A trial of the ECOG-ACRIN Cancer Research Group (EA5181). JCO. (2021) 39:TPS8584–TPS8584. doi: 10.1200/JCO.2021.39.15_suppl.TPS8584

[78] Ohri N Jolly S Cooper BT Kabarriti R Bodner WR Klein J . Selective personalized radioImmunotherapy for locally advanced non–small-cell lung cancer trial (SPRINT). JCO. (2024) 42:562–70. doi: 10.1200/JCO.23.00627, PMID: 37988638

[79] Liveringhouse CL Latifi K Asous AG Lam NB Rosenberg SA Dilling TJ . Dose-limiting pulmonary toxicity in a phase 1/2 study of radiation and chemotherapy with ipilimumab followed by nivolumab for patients with stage 3 unresectable non-small cell lung cancer. Int J Radiat OncologyBiologyPhysics. (2023) 116:837–48. doi: 10.1016/j.ijrobp.2023.01.006, PMID: 36657497 PMC12829912

[80] Barlesi F Cho BC Goldberg SB Yoh K Zimmer Gelatti AC Mann H . PACIFIC-9: Phase III trial of durvalumab + oleclumab or monalizumab in unresectable stage III non-small-cell lung cancer. Future Oncol. (2024) 20:2137–47. doi: 10.1080/14796694.2024.2354160, PMID: 39023287 PMC11508940

[81] Raben D Rimner A Senan S Broadhurst H Pellas T Dennis PA . Patterns of disease progression with durvalumab in stage III non-small cell lung cancer (PACIFIC). Int J Radiat OncologyBiologyPhysics. (2019) 105:683. doi: 10.1016/j.ijrobp.2019.08.034

[82] Berman A Chao HH Simone C Kegelman T Aggarwal C Bauml J . Long-term outcomes of proton beam re-irradiation for locoregionally recurrent non-small cell lung cancer. Int J Radiat OncologyBiologyPhysics. (2020) 108:E13–4. doi: 10.1016/j.ijrobp.2020.02.495

[83] Yegya-Raman N Berman AT Ciunci CA Friedes C Berlin E Iocolano M . Phase 2 trial of consolidation pembrolizumab after proton reirradiation for thoracic recurrences of non-small cell lung cancer. Int J Radiat OncologyBiologyPhysics. (2023) 119:56–65. doi: 10.1200/JCO.2023.41.16_suppl.8552, PMID: 37652303

[84] Yegya-Raman N Berman AT Ciunci CA Wang X Friedes C Iocolano M . Phase II trial of consolidation pembrolizumab (pembro) after proton reirradiation (re-RT) for thoracic recurrences of non-small cell lung cancer (NSCLC). JCO. (2023) 41:8552–2. doi: 10.1200/JCO.2023.41.16_suppl.8552, PMID: 37652303

[85] Ganti AK Klein AB Cotarla I Seal B Chou E . Update of incidence, prevalence, survival, and initial treatment in patients with non–small cell lung cancer in the US. JAMA Oncol. (2021) 7:1824. doi: 10.1001/jamaoncol.2021.4932, PMID: 34673888 PMC8532041

[86] Mazieres J Drilon A Lusque A Mhanna L Cortot AB Mezquita L . Immune checkpoint inhibitors for patients with advanced lung cancer and oncogenic driver alterations: results from the IMMUNOTARGET registry. Ann Oncol. (2019) 30:1321–8. doi: 10.1093/annonc/mdz167, PMID: 31125062 PMC7389252

[87] Galassi C Chan TA Vitale I Galluzzi L . The hallmarks of cancer immune evasion. Cancer Cell. (2024) 42:1825–63. doi: 10.1016/j.ccell.2024.09.010, PMID: 39393356

[88] Theelen WSME Peulen HMU Lalezari F van der Noort V De Vries JF Aerts JGJV . Effect of pembrolizumab after stereotactic body radiotherapy vs pembrolizumab alone on tumor response in patients with advanced non–small cell lung cancer: results of the PEMBRO-RT phase 2 randomized clinical trial. JAMA Oncol. (2019) 5:1276. doi: 10.1001/jamaoncol.2019.1478, PMID: 31294749 PMC6624814

[89] Welsh J Menon H Chen D Verma V Tang C Altan M . Pembrolizumab with or without radiation therapy for metastatic non-small cell lung cancer: a randomized phase I/II trial. J Immunother Cancer. (2020) 8:e001001. doi: 10.1136/jitc-2020-001001, PMID: 33051340 PMC7555111

[90] Mattes MD Eubank TD Almubarak M Wen S Marano GD Jacobson GM . A prospective trial evaluating the safety and systemic response from the concurrent use of radiation therapy with checkpoint inhibitor immunotherapy in metastatic non–small cell lung cancer. Clin Lung Cancer. (2021) 22:268–73. doi: 10.1016/j.cllc.2021.01.012, PMID: 33608212 PMC8310528

[91] Horndalsveen H Alver TN Dalsgaard AM Rogg LV Helbekkmo N Grønberg BH . Atezolizumab and stereotactic body radiotherapy in patients with advanced non-small cell lung cancer: safety, clinical activity and ctDNA responses—the ComIT-1 trial. Mol Oncol. (2023) 17:487–98. doi: 10.1002/1878-0261.13330, PMID: 36330681 PMC9980306

[92] Formenti SC Rudqvist NP Golden E Cooper B Wennerberg E Lhuillier C . Radiotherapy induces responses of lung cancer to CTLA-4 blockade. Nat Med. (2018) 24:1845–51. doi: 10.1038/s41591-018-0232-2, PMID: 30397353 PMC6286242

[93] Xue J Zhou X Zhou L Huang M Gong Y Zou B . Safety and efficacy of sintilimab in combination with SBRT and LDRT in PD-L1 positive treatment naïve-stage IV non-small cell lung cancer: A phase I study. JCO. (2022) 40:e21174–4. doi: 10.1200/JCO.2022.40.16_suppl.e21174

[94] Bestvina CM Pointer KB Karrison T Al-Hallaq H Hoffman PC Jelinek MJ . A phase 1 trial of concurrent or sequential ipilimumab, nivolumab, and stereotactic body radiotherapy in patients with stage IV NSCLC study. J Thorac Oncol. (2022) 17:130–40. doi: 10.1016/j.jtho.2021.08.019, PMID: 34500113

[95] Hellmann MD Paz-Ares L Bernabe Caro R Zurawski B Kim SW Carcereny Costa E . Nivolumab plus ipilimumab in advanced non–small-cell lung cancer. N Engl J Med. (2019) 381:2020–31. doi: 10.1056/NEJMoa1910231, PMID: 31562796

[96] Brahmer JR Lee JS Ciuleanu TE Bernabe Caro R Nishio M Urban L . Five-year survival outcomes with nivolumab plus ipilimumab versus chemotherapy as first-line treatment for metastatic non–small-cell lung cancer in checkMate 227. JCO. (2023) 41:1200–12. doi: 10.1200/JCO.22.01503, PMID: 36223558 PMC9937094

[97] Mahase SS Golden EB Lin E Saxena A Scheff RJ Ballman K . Radiation and immune checkpoints blockade in metastatic NSCLC. Int J Radiat OncologyBiologyPhysics. (2020) 108:e93–4. doi: 10.1016/j.ijrobp.2020.07.1197

[98] Schoenfeld JD Giobbie-Hurder A Ranasinghe S Kao KZ Lako A Tsuji J . Durvalumab plus tremelimumab alone or in combination with low-dose or hypofractionated radiotherapy in metastatic non-small-cell lung cancer refractory to previous PD(L)-1 therapy: an open-label, multicentre, randomised, phase 2 trial. Lancet Oncol. (2022) 23:279–91. doi: 10.1016/S1470-2045(21)00658-6, PMID: 35033226 PMC8813905

[99] PPD . Challenges and opportunities in clinical trials (2022). Wilmington, NC: PPD. Available online at: https://www.ppd.com/blog/challenges-opportunities-in-clinical-trials/ (Accessed March 15, 2025).

[100] PD . Drug development challenges and opportunities in 2024 (2024). Wilmington, NC: PPD. Available online at: https://www.ppd.com/blog/drug-development-challenges-opportunities-2024/ (Accessed March 15, 2025).

[101] Cucurull-Sanchez L . An industry perspective on current QSP trends in drug development. J Pharmacokinet Pharmacodyn. (2024) 51:511–20. doi: 10.1007/s10928-024-09905-y, PMID: 38443663 PMC11576823

[102] Sové RJ Jafarnejad M Zhao C Wang H Ma H Popel AS . QSP-IO: A quantitative systems pharmacology toolbox for mechanistic multiscale modeling for immuno-oncology applications. Clin Pharmacol Ther. (2020) 9:484–97. doi: 10.1002/psp4.12546, PMID: 32618119 PMC7499194

[103] Schirru M Charef H Ismaili KE Fenneteau F Zugaj D Tremblay PO . Predicting efficacy assessment of combined treatment of radiotherapy and nivolumab for NSCLC patients through virtual clinical trials using QSP modeling. J Pharmacokinet Pharmacodyn. (2024) 51:319–33. doi: 10.1007/s10928-024-09903-0, PMID: 38493439

[104] Chen DS Mellman I . Oncology meets immunology: the cancer-immunity cycle. Immunity. (2013) 39:1–10. doi: 10.1016/j.immuni.2013.07.012, PMID: 23890059

[105] Ye H Pang H Shi X Ren P Huang S Yu H . Nivolumab and hypofractionated radiotherapy in patients with advanced lung cancer: ABSCOPAL-1 clinical trial. Front Oncol. (2021) 11:657024. doi: 10.3389/fonc.2021.657024, PMID: 33968760 PMC8100893

[106] Charef H Schirru M Ismaili K-E Fenneteau F Zugaj D Tremblay P-O . Implementation and evaluation of radiotherapy combined with anti-PD-1 treatment in a virtual population of patients with non-small cell lung cancer (2023). Bellevue, WA: Abstracts of the Fourteenth American Conference on Pharmacometrics (ACoP14. Available online at: https://online.fliphtml5.com/gbngh/wata/p=96 (Accessed March 15, 2025).

[107] Balti A Zugaj D Fenneteau F Tremblay PO Nekka F . Dynamical systems analysis as an additional tool to inform treatment outcomes: The case study of a quantitative systems pharmacology model of immuno-oncology. Chaos: Interdiscip J Nonlinear Science. (2021) 31:023124. doi: 10.1063/5.0022238, PMID: 33653032

[108] Zugaj D Fenneteau F Tremblay PO Nekka F . Dynamical behavior-based approach for the evaluation of treatment efficacy: The case of immuno-oncology. Chaos: Interdiscip J Nonlinear Science. (2024) 34:013142. doi: 10.1063/5.0170329, PMID: 38277131

[109] Khalifa J Mazieres J Gomez-Roca C Ayyoub M Moyal ECJ . Radiotherapy in the era of immunotherapy with a focus on non-small-cell lung cancer: time to revisit ancient dogmas? Front Oncol. (2021) 11:662236. doi: 10.3389/fonc.2021.662236, PMID: 33968769 PMC8097090

[110] Forde PM Spicer J Lu S Provencio M Mitsudomi T Awad MM . Neoadjuvant nivolumab plus chemotherapy in resectable lung cancer. N Engl J Med. (2022) 386:1973–85. doi: 10.1056/NEJMoa2202170, PMID: 35403841 PMC9844511

[111] Provencio M Nadal E González-Larriba JL Martínez-Martí A Bernabé R Bosch-Barrera J . Perioperative nivolumab and chemotherapy in stage III non–small-cell lung cancer. N Engl J Med. (2023) 389:504–13. doi: 10.1056/NEJMoa2215530, PMID: 37379158

[112] Heymach JV Harpole D Mitsudomi T Taube JM Galffy G Hochmair M . Perioperative durvalumab for resectable non–small-cell lung cancer. N Engl J Med. (2023) 389:1672–84. doi: 10.1056/NEJMoa2304875, PMID: 37870974

[113] Cascone T Awad MM Spicer JD He J Lu S Sepesi B . Perioperative nivolumab in resectable lung cancer. N Engl J Med. (2024) 390:1756–69. doi: 10.1056/NEJMoa2311926, PMID: 38749033

[114] Felip E Altorki N Zhou C Csőszi T Vynnychenko I Goloborodko O . Adjuvant atezolizumab after adjuvant chemotherapy in resected stage IB–IIIA non-small-cell lung cancer (IMpower010): a randomised, multicentre, open-label, phase 3 trial. Lancet. (2021) 398:1344–57. doi: 10.1016/S0140-6736(21)02098-5, PMID: 34555333

